# Comprehensive characterization of the RNA editing landscape in the human aging brains with Alzheimer's disease

**DOI:** 10.1002/alz.70452

**Published:** 2025-07-09

**Authors:** Amit Kumar Gupta, William Martin, Andrew A. Pieper, Yinsheng Wang, Andrew J. Saykin, Feixiong Cheng

**Affiliations:** ^1^ Cleveland Clinic Genome Center Lerner Research Institute Cleveland Clinic Cleveland Ohio USA; ^2^ Genomic Medicine Institute Lerner Research Institute Cleveland Clinic Cleveland Ohio USA; ^3^ Department of Psychiatry Case Western Reserve University Cleveland Ohio USA; ^4^ Brain Health Medicines Center Harrington Discovery Institute University Hospitals Cleveland Medical Center Cleveland Ohio USA; ^5^ Geriatric Psychiatry GRECC Louis Stokes Cleveland VA Medical Center Cleveland Ohio USA; ^6^ Institute for Transformative Molecular Medicine School of Medicine Case Western Reserve University Cleveland Ohio USA; ^7^ Department of Pathology Case Western Reserve University School of Medicine Cleveland Ohio USA; ^8^ Department of Neurosciences Case Western Reserve University School of Medicine Cleveland Ohio USA; ^9^ Department of Chemistry University of California Riverside California USA; ^10^ Environmental Toxicology Graduate Program University of California Riverside California USA; ^11^ Department of Radiology and Imaging Sciences Center for Neuroimaging Indiana University School of Medicine Indianapolis Indiana USA; ^12^ Indiana Alzheimer's Disease Research Center Indiana University School of Medicine Indianapolis Indiana USA; ^13^ Department of Medical and Molecular Genetics Indiana University School of Medicine Indianapolis Indiana USA; ^14^ Center for Computational Biology and Bioinformatics Indiana University School of Medicine Indianapolis Indiana USA; ^15^ Department of Molecular Medicine Cleveland Clinic Lerner College of Medicine Case Western Reserve University Cleveland Ohio USA; ^16^ Case Comprehensive Cancer Center Case Western Reserve University School of Medicine Cleveland Ohio USA

**Keywords:** Alzheimer's disease, biomarkers, drug target, genome regulatory network, quantitative trait loci (QTL), RNA editing

## Abstract

**INTRODUCTION:**

While RNA editing has been linked to Alzheimer's disease (AD), its specific impact on the transcriptomic landscape in human AD brains remains under explored.

**METHODS:**

We conducted a comprehensive analysis of RNA editing across nine human brain regions affected by AD, utilizing RNA‐seq data and matched whole‐genome sequencing data from three human brain biobanks, adjusting for age, *post mortem* interval, sex, and apolipoprotein E4 (*APOE4)* status.

**RESULTS:**

RNA‐editing events were identified in both AD and healthy control aging brains, highlighting 127 genes with significant RNA editing loci. AD exhibited elevated RNA editing in the parahippocampal gyrus and cerebellar cortex. We also discovered 147 colocalized genome‐wide association studies (GWAS) and *cis*‐edQTL (± 1 MB) signals in 48 likely causal genes encompassing *CLU*, *BIN1*, and *GRIN3B*, primarily allied to amyloid and tau pathology, and neuroinflammation.

**DISCUSSION:**

Our findings delineate RNA editing regulatory signatures in human AD, providing novel insights into AD pathophysiology and potential biomarkers and therapeutic targets.

**Highlights:**

· We discovered genome‐wide landscape of RNA editing signals from 4208 (1364 Alzheimer's disease [AD] cases vs. 742 healthy controls) RNA‐seq data across nine human brain regions from three large brain biobanks (Mount Sinai Brain Bank [MSBB], Mayo Clinic [MAYO] Religious Order Study and Memory and Aging Project [ROSMAP]) tied with AD, including in sex‐specific and apolipoprotein E4 (APOE4) ‐specific manner adjusting for age, *post mortem* interval (PMI), sex, and APOE4 status.
· We emphasize 127 genes, including *SYT11*, *KCNIP4*, *NRG3*, *ANKS1B*, and *RALYL*, exhibiting significant RNA editing loci shared by multiple brain tissues, mainly implicated in synaptic plasticity, signaling and transmission, neuronal development, and morphogenesis.
· Brain‐wide tissue‐specific *cis*‐regulatory variants (*cis*‐edQTLs) were inspected using matched genotyping data from 3627 samples from all brain biobanks. We revealed 147 colocalized AD‐GWAS and *cis*‐edQTLs signals pertaining to 48 likely causal genes comprising *CLU* (rs7982, rs1532278), *BIN1* (rs2276582, rs3768863), *GRIN3B* (rs10417824, rs1058603), *NYAP1* (rs12539172), *DGKQ* (rs4690197, rs3733347), *CLPTM1* (rs204468), etc.
· Colocalized signals show affiliations to tau protein binding, amyloid‐β regulation, cellular morphogenesis, and immune response pathway suggesting possible roles of epitranscriptomic mechanisms in shaping the AD risk.

## 1 BACKGROUND

RNA editing is a post‐transcriptional modification and an established molecular process through which cells make edits in the RNA transcripts, including insertion, deletion, and base substitution of nucleotides within different types of RNA molecules (messenger RNA [mRNA], transfer RNA [tRNA], ribosomal RNA [rRNA], microRNA [miRNA]).^1^, ^2^ It plays a vital role in the diversification of protein isoforms (protein diversity) and can affect various cellular functions such as RNA stability, localization, splicing, abundance, structure, and translation.^2^, ^3^ Although it is rare, RNA editing is a critical mechanism for generating transcriptomic diversity,^3^ predominantly involving two types of modifications: A‐to‐I (adenosine to inosine) and C‐to‐U (cytidine to uridine) transition. This process is regulated by various factors, such as genetic loci and RNA binding protein editing regulators.^4^, ^5^ Notably, RNA editing can alter both protein coding and noncoding regions,^6^ and cause protein recoding (codon change), mRNA/protein degradation, alternative RNA splicing, gene silencing, miRNA processing, and RNA binding.^2^, ^5^, ^7^ It is gradually being known to be associated with the plethora of biological functions with immune defense and neuronal regulations.^1^, ^2^, ^8^ RNA editing has been implicated in various conditions, particularly in cancer and Alzheimer's disease (AD).^9^, ^10^, ^11^

AD, the most prevalent form of age‐related dementia, currently affects approximately 6.7 million Americans, with projections indicating that this number could reach 14 million by 2060.^12^ The pathophysiology of AD remains poorly understood, and effective treatments are lacking. Genetic factors, such as mutations in the genes for apolipoprotein E (APOE) Ɛ4 or β‐amyloid,^13^, ^14^, ^15^ contribute to AD risk, but environmental factors and biological variables also play important role in the trajectory of AD. These include comorbid diseases (i.e., diabetes mellitus, depression, hypercholesterolemia, hypertension), environmental factors (i.e., pesticide exposure, smoking, air pollution, and traumatic brain injury),^16^, ^17^, ^18^, ^19^ and biological variables (i.e., sex, age, and ethnicity).^20^, ^21^ Moreover, genetic/genomic variations in multiple genes (*APP, PSEN1, PSEN2, BIN1, CLU, ABCA7, CR1*, and *PICALM*) also generate a complex genetic architecture that generates a wide spectrum of AD disease.^13^, ^22^, ^23^

**RESEARCH IN CONTEXT**

1. **Systematic review**: RNA editing represents the most common post‐transcriptional modifications contributing to transcriptomic diversity, RNA stability, and alternative splicing, impacting immune response and neuronal dynamics. While RNA editing remains of great interest and has been linked to neurological conditions including Alzheimer's disease (AD), the genome‐wide tissue specific landscape and its impact on the epitranscriptomic regulations in human AD brains remain relatively underexplored. In this study, we investigated the large‐scale human brain biobanks to characterize brain region‐specific editing signatures associated with AD with sex and apolipoprotein E4 (*APOE4)* ‐specificity, brain‐wide *cis*‐regulations, and discovered likely causal relationship utilizing AD genome‐wide association study (GWAS) summary statistics.
2. **Interpretation**: We have uncovered genome‐wide landscape of tissue‐specific RNA editing signals across nine brain regions through leveraging large‐scale RNA‐seq and whole‐genome sequencing (WGS) data from three large brain biobanks (MSBB [Mount Sinai Brain Bank], MAYO [Mayo Clinic], Religious Order Study and Memory and Aging Project [ROSMAP]). We accentuate 127 genes, including *SYT11*, *KCNIP4*, *NRG3*, *ANKS1B*, and *RALYL*, exhibiting significant RNA editing loci shared among several brain tissues, mainly associated to synaptic plasticity, signaling and transmission, neuronal development, and morphogenesis. We also report 147 colocalized AD‐GWAS and *cis*‐edQTLs signals relating to 48 likely causal genes comprising *CLU* (rs7982, rs1532278), *BIN1* (rs2276582, rs3768863), *GRIN3B* (rs10417824, rs1058603), *NYAP1* (rs12539172), *DGKQ* (rs4690197, rs3733347), and *CLPTM1* (rs204468), mainly implicated in tau protein binding, amyloid‐β regulation, cellular morphogenesis, and immune response pathway, suggesting possible role of epitranscriptomic mechanism in the disease pathology.
3. **Future directions**: Our findings demonstrate the significance of tissue‐specific RNA editing and regulatory signatures in human AD, highlighting its role in neuronal and synaptic regulation. Essentially, we augment understanding of RNA edited targets and mechanism implicated in disease risk that could pave the way for identifying potential biomarkers and therapeutics in AD and AD‐related dementias if broadly applied.

Despite extensive genome‐wide association studies (GWAS) identifying numerous candidate genes and genomic loci associated with AD,^24^, ^25^, ^26^, ^27^, ^28^, ^29^ as well as myriad epigenetic changes in AD,^3^, ^30^, ^31^, ^32^ the role of RNA editing remains of great interest by comparison relatively unexplored.^33^, ^34^, ^35^, ^36^ Indeed, characterizing RNA editing in the human AD brain could enhance our understanding of AD pathophysiology and reveal new biomarkers and therapeutic targets.^2^, ^37^, ^38^

In this study, we comprehensively characterize genome‐wide RNA editing events in large, harmonized cohorts of AD, compared to matched healthy controls (HCs). We identify RNA editing events associated with AD, as well as sex‐specific and *APOE4*‐specific altered editing signals. We also report the landscape of brain‐wide *cis* acting genetic variants, such as RNA editing quantitative trait loci (*cis*‐edQTL), that accentuate the complexity of RNA editing‐mediated regulation. Our results augment understanding of RNA edited target genes and mechanism implicated in disease risk, paving the way for future RNA‐based biomarkers and therapeutics.

## 2 METHODS

### 2.1 RNAseq data processing and mapping

#### 2.1.1 MSBB Biobank

In total, 1247 MSBB (Mount Sinai Brain Bank) single‐end RNA‐seq sample bam files across four different brain regions (frontal pole (FP) [*n* = 318], superior temporal gyrus (STG) [*n* = 324], parahippocampal gyrus (PHG) [*n* = 308], inferior frontal gyrus (IFG) [*n* = 297]) were converted into fastq files utilizing samtools (v1.16.1).^39^ These fastq files and corresponding available unmapped fastq were merged. The merged fastq file was mapped using STAR (v2.7.10a) mapper^40^ against STAR index human GRCh38 genome assembly, spending multi threads on high performance computing (HPC). Human genome (hg38) aligned bam files were sorted and indexed using samtools.^39^

#### 2.1.2 MAYO Biobank

Five hundred five MAYO (Mayo Clinic) paired‐end RNA‐seq sample bam files from two brain regions, that is, cerebellum cortex (CBE) (*n* = 246) and temporal cortex (TCX) (*n* = 259) were processed to obtained forward (R1) and reverse (R2) read fastq files with samtools. Paired end fastq files were mapped with STAR and indexed using samtools, as described above.

#### 2.1.3 ROSMAP Biobank

Two thousand four hundred fifty‐six ROSMAP (Rush University's Religious Orders Study and Memory and Aging Project) paired‐end RNA‐seq samples from three brain regions (dorsolateral prefrontal cortex [DLPFC], 1,092; anterior cingulate cortex [ACC], 717, and posterior cingulate cortex [PCC], 647) were processed. Two thousand one hundred thirty‐one RNAseq sample bam files were processed to generate forward and reverse read fastq files using samtools. However, 325 raw paired end fastq files (batch 3 data) were first subjected to quality check (QC) filtering employing fastp 0.23.2 tool^41^ with default setting except qualified Phred score (≥Q20) and allowed an unqualified percent bases limit (30%). All the samples were mapped to the human genome (hg38) with STAR mapper followed by sorting and indexing using samtools.^39^

### 2.2 Whole‐genome sequencing data processing, filtering, and annotation

Genotype data from all three biobanks (MSBB, MAYO, ROSMAP) were first subjected to the QC utilizing the PLINK v2.00a2LM tool.^42^, ^43^ Filtering criteria used for QC were missing genotypes (0.01), duplicates removal, single‐nucleotide polymorphisms (SNPs) only, Bi‐allelic SNPs, minimum allele frequency (MAF) (0.05), and Hardy–Weinberg equilibrium (HWE) (1e‐6). Assembly conversion was applied to convert the genotype data from hg19 to hg38 using CrossMap (v0.6.6)^44^ with hg19ToHg38 chain file. The converted variant call format (VCF) files were further processed using vcftools (0.1.15),^45^ bcftools (1.16),^46^ and tabix (htslib/1.16).^47^ First, VCFs were sorted using vcf‐sort (vcftools/0.1.15) and bgzip (htslib/1.16) compressed. Then, annotated employing dbSNP 156 utilizing bcftools annotate was applied and indexed using tabix (htslib/1.16) function. This annotated genotype was later utilized for *cis* RNA editing QTL (*cis*‐edQTL) identification.

### 2.3 RNA editing identification and association analysis

Genome‐wide brain‐region specific RNA editing sites were identified utilizing REDItools^48^, ^49^, ^50^ 2 on HPC with default settings using mpirun for parallelization. All RNAseq bam files from the mapping step were subjected to chromosome‐wide coverage calculation implementing samtools depth function through REDItools using human genome hg38 assembly. Next, sample‐wise RNA editing sites and frequency was quantified and merged to generate frequency matrix using REDItools 2 (python 2.7 and pysam 0.15.3). Later, RNA editing selection and filtering were performed using selectPositions module from REDItools 2 with default parameters, that is, read coverage,^5^ variation bases,^1^ variation frequency [0.1], and substitutions [all].

Distinct RNA editing frequency matrices and phenotype tables were compiled for the AD+HC samples for each brain region (215 FP, 226 PHG, 220 STG, 201 IFG, 144 CBE, 148 TCX, 437 DLPFC, 250 PCC, and 265 ACC). RNA editing sites were removed from the merged editing table if the site was missing in 90% of the samples for each brain region. These filtered RNA editing tables were used to perform RNA editing association analysis in R v4.3.2 environment using the rnaEditr (1.10.0) package (https://bioconductor.org/packages/rnaEditr). Multiple steps were followed to perform the analysis. Briefly, GRanges object was defined as storing hg38 annotated 19,924 genes to provide input genome region utilizing the rnaEditr package. Then, “AllCloseByRegions” function was used to extract clusters (maxGap: 50 bp, minSites: 3) of RNA editing sites located closely in the defined genomic regions. Every resulting cluster (close‐by region) included a group of genomic sites within 50 bp (maxGap) from each other and a minimum of three RNA editing sites (minSites) in each. Next, co‐edited regions within each close‐by‐region (cluster) were identified based on RNA editing levels using “AllCoeditedRegions” function. Criteria to define co‐edited region were at least three editing sites (minSites), minimum correlation between RNA editing level of one site with the rest of the sites (rDropThresh: 0.4), and minimum pairwise correlation (Spearman) of sites within the cluster of 0.1 (minPairCorr). RNA editing levels were further summarized from multiple sites by taking median (MedianSites) utilizing the SummarizeAllRegions function. Lastly, association analysis was performed using TestAssociations function between phenotype (AD vs. HC) and region‐based summarized RNA editing levels, adjusting with covariates (*APOE4*, sex, *post mortem* interval [PMI], and age). Similarly, RNA editing association analysis between ADfemale versus ADmale and ADapoe4 versus ADnon‐apoe4 was also performed. Association analysis results were annotated with human genome (hg38) by AnnotateResults function (rnaEditr).

Later, association results were depicted through the Manhattan plots highlighting significant genes (*p* ≤ 0.0005) by CMplot package^51^ in R. Gene ontology and pathway enrichment analysis was performed for the RNA editing associated set of genes from each brain region using ShinyGO 0.77 (0.80),^52^, ^53^ g:Profiler^54^ ve110_eg57_p18_4b54a898, and Enrichr.^55^, ^56^ The RNA editing landscape from different brain regions was illustrated utilizing the circlize 0.4.15 package^57^ in R, which marked AD‐associated genes harboring significant RNA editing loci (*p*‐value 5_*_10^−3^) shared by at least two brain regions. Distinct R packages, that is, tidyverse (2.0.0), ggplot2 (3.4.2) (https://ggplot2.tidyverse.org/), and smplot2 (0.1.0),^58^ were used for visualization.

### 2.4 Brain‐wide *cis*‐edQTLs

Brain‐wide *cis* RNA editing QTL (*cis*‐edQTLs) were identified using MatrixEQTL (v2.3) package^59^ in R and GTEx_edQTL^60^ (https://github.com/vargasliqin/GTEx_edQTL/) repository from Github. We compiled RNA editing matrices of matched RNAseq and whole‐genome sequencing (WGS) samples for nine brain regions (FP, 302 samples; PHG, 292; STG, 308; IFG, 281; CBE, 243; TCX, 255; DLPFC, 821; PCC, 496; and ACC, 629). Brain‐region specific individual sample RNA editing data files were combined into a matrix utilizing the sharedsamples_sites_matrix_FastQTL_v8.pl (GTEx_edQTL) perl script with setting the minimum number of samples per site (minsamps: 10 samples) and minimum reads coverage per site (mincov: 10 reads). The resulting editing level matrices were further processed to prepare a phenotype table with quantile normalization across samples using prepare_phenotype_table_for_QTLtools.V8.py (GTEx_edQTL) script. Brain‐region specific genotype (VCF) files were prepared utilizing the annotated genotype (hg38) data from the three biobanks (MSBB, MAYO, ROSMAP). These RNA editing phenotype files and corresponding genotype data from nine brain regions was used for the *cis*‐edQTL identification via MatrixEQTL (v2.3) package.^59^ We performed *cis*‐edQTL identification at two distances for each brain region, that is, ± 100 KB and ± 1 MB with significance threshold of 0.05.

### 2.5 Colocalization and downstream analysis

Significant AD GWAS loci (false discovery rate [FDR] ≤ 5×10^−2^) from the Bellenguez et al.^24^ and *cis*‐edQTL sites from each brain region were used for colocalization analysis. GWAS and edQTL summary statistics were utilized with coloc (Bayesian function)^61^, ^62^, ^63^ and posterior probabilities (PP) were calculated. Loci with PP4 greater than 0.5 were contemplated as colocalized GWAS and edQTL signals. Chromosome‐wise colocalized signatures were plotted utilizing the LocusCompareR^64^ and locuszoom^65^ packages. Identified target genes harboring GWAS‐edQTL loci were subjected to downstream regulatory analysis to identify associated transcription factors (TFs) and miRNAs employing NetworkAnalyst 3.0^66^ utilizing TRRUST,^67^ miRTarBase v8.0,^68^ and RegNetwork.^69^ Regulatory network was visualized using the cytoscape v3.9.1.^70^

## 3 RESULTS

### 3.1 Landscape of RNA editing in the human aging brain with AD

**FIGURE 1:**
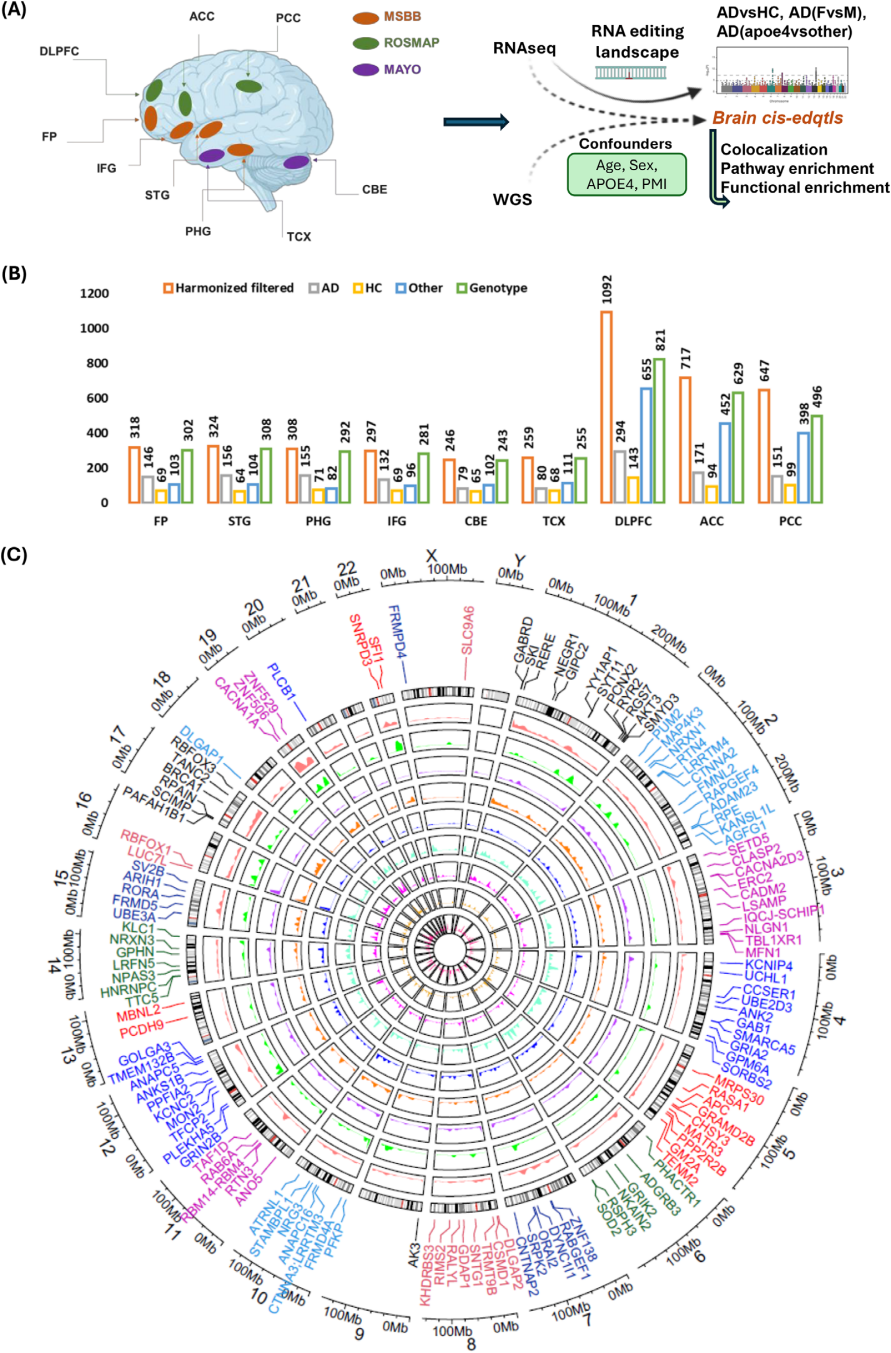
(A) Overview of the study. (B) Brain region‐specific sample distribution. (C) Circular plot depicting the brain region‐specific landscape of RNA editing (*cf*. Methods) events. Nine circular tracks show editing level and density from different brain regions (CBE, TCX, PHG, IFG, STG, FP, DLPFC, PCC, ACC). The plot highlights 127 genes harboring significant RNA editing loci (*p* ≤ 5×10‐3) that are shared by two or more brain regions. ACC, anterior cingulate cortex; AD, Alzheimer's disease; CBE, cerebellum cortex; DLPFC, dorsolateral prefrontal cortex; FP, frontal pole; HC, healthy controls; IFG, inferior frontal gyrus; MAYO, Mayo Clinic; MSBB, Mount Sinai Brain Bank; PCC, posterior cingulate cortex; PHG, parahippocampal gyrus; ROSMAP, Rush University's Religious Orders Study and Memory and Aging Project; STG, superior temporal gyrus; TCX, temporal cortex.

Brain region‐specific transcriptomic (bulk RNA‐seq) and WGS data were collected from both Synapse (https://www.synapse.org) and the RNAseq harmonization study (Synapse ID: syn21241740). These data comprised three major cohorts from the AMP‐AD consortium: MSBB, MAYO, and ROSMAP, all of which contain AD and HC subjects (Figure 1A). The MSBB cohort (Synapse ID: syn3159438) consists of 318 FP, 324 STG, 308 PHG, and 297 IFG samples. The MAYO cohort (Synapse ID: syn5550404) consists of 246 CBE and 259 TCX samples. The ROSMAP cohort (Synapse ID: syn3219045) consists of 1092 DLPFC, 717 ACC, and 647 PCC samples. Thus, we queried a total of 4208 RNAseq samples from nine brain regions, including 2106 AD+HC samples (Figure 1B). A summary of the region‐specific samples from each cohort is provided in Table S1. Many covariates were considered, including sex, *post mortem* interval (PMI), age, and *APOE4* status, utilizing available clinical metadata from the RNAseq harmonization study (syn21241740). Furthermore, RNAseq sample IDs from the AMP‐AD primary cohorts were matched with WGS metadata to yield matched WGS data for each brain region. The study workflow is illustrated in Figures 1A and S1.

To investigate AD‐associated RNA editing variants and gene regulation beyond the level of transcription, we also analyzed epitranscriptomic RNA editing events from different brain regions. We identified a large repertoire of RNA editing sites (454,585–1,416,230) across all nine brain regions in the total sample set, which were clustered to the edited regions for each brain tissues. The PHG exhibited the highest abundance (14,687; *p* ≤ 5×10^−2^), while TCX exhibited the lowest (1800; *p* ≤ 5×10^−2^) (Tables S2–S10). These RNA editing sites were mainly enriched in intronic genomic regions, followed by exons and 3'‐ untranslated regions (3'UTRs) (Figures S2–S4). Thus, we identified genome‐wide AD‐associated RNA editing alterations in sex‐specific or *APOE4*‐specific manners, as well as likely causal genetic variants and genes associated with AD that may regulate cellular function.

### 3.2 A genome regulatory map of RNA editing events in human AD brains

**FIGURE 2:**
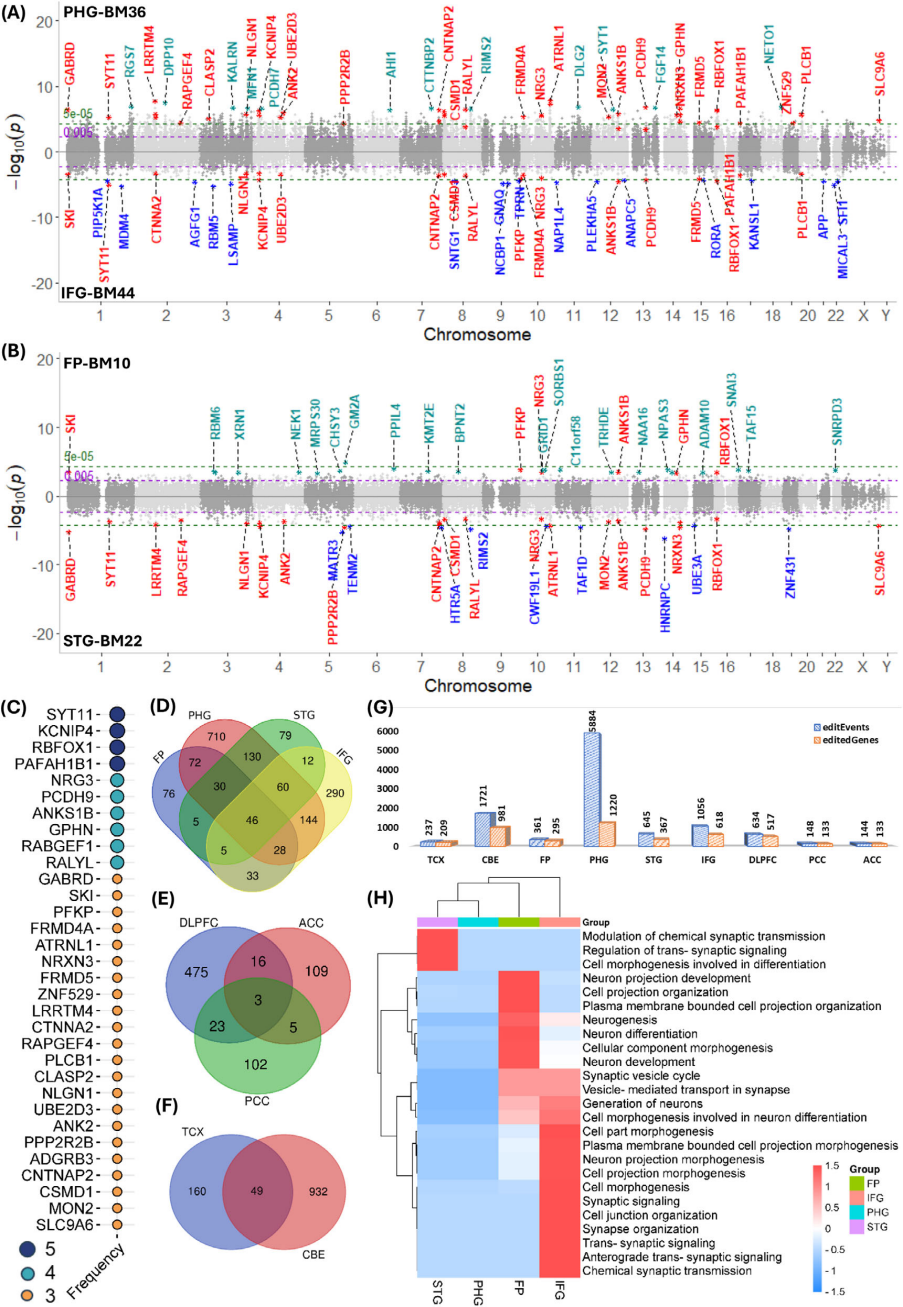
Manhattan plots illustrating the distribution of genome wide RNA editing events and significant RNA editing genes from four MSBB brain regions: (A) PHG and IFG, (B) FP and STG, respectively; (C) Significantly associated RNA edited genes in at least three brain tissues; Number of shared AD‐associated RNA edited genes between distinct brain regions from three biobanks (D) MSBB, (E) ROSMAP, (F) MAYO; (G) Number of editing events and genes across brain regions; (H) Functional enrichment of RNA edited genes from four MSBB brain regions (FP, STG, PHG, IFG). AD, Alzheimer's disease; FP, frontal pole; IFG, inferior frontal gyrus; MAYO, Mayo Clinic; MSBB, Mount Sinai Brain Bank; PHG, parahippocampal gyrus; ROSMAP, Rush University's Religious Orders Study and Memory and Aging Project; STG, superior temporal gyrus.

We next explored AD‐associated RNA edited regions (clusters) across brain regions to identify associated target genes and discovered 127 genes harboring significant RNA edited loci (*p* ≤ 5×10^−3^) that were shared by two or more brain regions (Figure 1C). Of note, the PHG and the CBE exhibited the highest abundance of RNA editing events, followed by the IFG and DLPFC. Mirrored Manhattan plots show the distribution of genome wide RNA editing events and significant RNA edited genes from four MSBB brain regions, namely PHG and IFG (Figure 2A), and FP and STG (Figure 2B), and identified overlapping RNA edited genes between multiple brain regions from the three different biobanks (Figure 2C–F). This revealed RNA editing events and genes specific to distinct brain regions (Tables S2–S10). Genome‐wide distribution of these editing events from individual brain regions is also shown by Manhattan plot (Figures S5–S7). Overall, we discovered an array of brain region‐specific RNA‐edited genes, that is, 295 FP, 367 STG, 1220 PHG, 618 IFG, 517 DLPFC, 133 PCC, 133 ACC, 209 TCX, and 981 CBE (Figure 2G). Functional enrichment analysis of these genes is depicted in Figures S8–S10. Gene ontology and pathway analysis of region‐specific RNA edited genes broadly identified enrichment in neurogenesis, neuron development, synaptic function, and morphogenesis (Figure 2H, Tables S11–S17).

Notably, the majority of AD‐associated RNA edited genes were implicated in synaptic plasticity, signaling, and transmission, mainly including *SYT11*, *KCNIP4*, *NRG3*, *NRXN3*, *LRRTM4, NLGN1*, and *FRMD4A* (Figure 2C). *SYT11* (Synaptotagmin‐11) (1q22)‐associated RNA edits showed significant association (*p* ≤ 5×10^−3^) with AD across five brain regions. The synaptotagmin 11 protein regulates immune functions, synaptic plasticity, and parkin‐linked neurotoxicity^71^, ^72^, ^73^ and *SYT11* has been implicated in multiple neurological diseases, including AD, Parkinson's disease (PD), schizophrenia, autism, and epilepsy.^72^, ^74^, ^75^ Furthermore, depletion of *SYT11* is linked to lysosomal dysfunction, autophagy blockage, and α‐synuclein accumulation.^76^ The potassium (Kv) channel‐interacting protein 4 (KCNIP4) (4p15.2) has been implicated in attention‐deficit/hyperactivity disorder (ADHD),^77^ while *PCDH9* (Protocadherin 9) (13q21.32) is a transmembrane protein that mediates cell adhesion in neural tissues and neuronal synaptic junction signaling.^78^ The *FRMD4A* (FERM Domain Containing 4A) (10p13) gene regulates epithelial cells and adherens junctions and variations in *FRMD4A* are associated with AD.^79^ *NRXN3* (Neurexin 3) (14q24.3) and *NLGN1* (Neuroligin 1) (3q26.31) encode cell surface proteins involved in nervous system development, including synaptic function, interactions, and transmission.^80^, ^81^ Likewise, the *LRRTM4* (leucine rich repeat transmembrane neuronal 4) (2p12) gene is associated with schizophrenia and epilepsy and involved in glutamatergic synapses, regulation of synapse assembly, and presynaptic membrane organization.^82^ The *NRG3* (10q23.1) gene belongs to the family of genes encoding neuregulin growth factors, which have been implicated in aberrant synaptic function and synaptogenesis in schizophrenia.^83^

We also identified several genes linked to neuronal morphogenesis, including *RBFOX1*, *RALYL*, *ATRNL1*, *CTNNA2*, *CNTNAP2*, *RABGEF1*, *GPHN*, *ANKS1B*, and *PAFAH1B1*. The RNA binding fox‐1 homolog 1 (*RBFOX1*) (16p13.3) gene regulates neuronal development,^84^ while *RALYL* (RALY RNA binding protein like) (8q21.2) has been only briefly studied in AD by network approach.^85^ The *ATRNL1* (attractin‐like 1) (10q25.3) gene is involved in G protein‐coupled receptor signaling, morphogenesis, and cell migration.^86^ The *CTNNA2* (catenin alpha 2) (2p12) gene regulate neuronal migration and projection development, as well as cell‐cell adhesion, differentiation, and synaptic plasticity in the nervous system.^87^ The *CNTNAP2* (contactin associated protein 2) gene (7q35) is a member of the neurexin family, which plays a crucial role in nervous system development as a cell adhesion and receptor molecule, and has been implicated in intellectual disability, epilepsy, schizophrenia, autism, and ADHD.^88^ The *RABGEF1* (RAB guanine nucleotide exchange factor 1) (7q11.21) encodes a protein that mediates endocytic membrane fusion,^89^ while the *GPHN* (gephyrin) (14q23.3) gene encodes a neuronal assembly protein involved in neurotransmitter‐synaptic microtubule interactions.^90^ The protein product of the *ANKS1B* (ankyrin repeat and sterile alpha motif domain containing 1B) (12q23.1) gene interacts with amyloid‐β precursor protein in AD pathogenesis.^91^ Lastly, the *PAFAH1B1* (platelet activating factor acetylhydrolase 1b regulatory subunit 1) (17p13.3) gene is essential for brain development, neuronal precursor proliferation, and neuronal migration.^92^

Moreover, we also identified an association of AD with significant RNA editing clusters in several other genes, including *GABRD*, *FRMD5*, *RAPGEF4*, and *ANK2*. *GABRD* (gamma‐aminobutyric acid type A receptor subunit delta) (1p36.33) mediates neuronal inhibition through GABA‐A receptor binding and is implicated in Akt signaling, chloride channel activity, and epilepsy.^93^, ^94^ *FRMD5* (FERM Domain Containing 5) (15q15.3), which has been implicated in neurodevelopment disorders, is involved in binding activities and regulating cell motility, adhesion, and migration.^95^ *RAPGEF4* (rap guanine nucleotide exchange factor 4) (2q31.1) regulates glutamatergic signaling and is associated with autism.^96^ Lastly, *ANK2* (ankyrin 2) (4q25) plays a key role in cell activation, proliferation, and motility in nervous system development and is associated with autism spectrum disorder.^97^

### 3.3 Sex‐specific differential RNA editing events in the human AD brain

**FIGURE 3:**
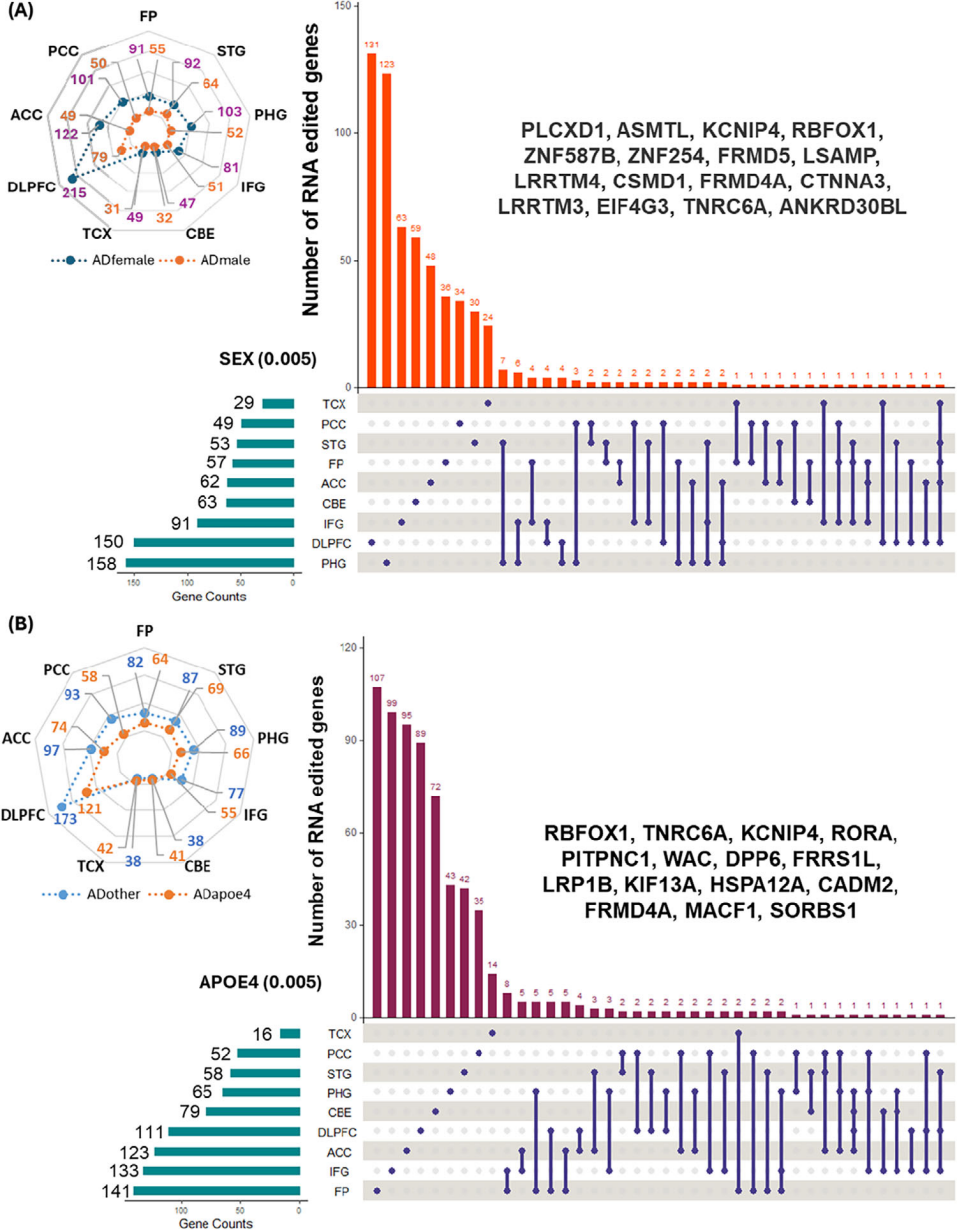
Radar plot for number of samples and upset plot for RNA edited genes in AD from individual brain regions for (A) sex‐specific (B) *APOE4*‐specific alterations. AD, Alzheimer's disease; *APOE4*, apolipoprotein E4.

Next, we investigated the sex‐specific patterns in RNA editing in individual brain regions. The group‐wise number of samples from each brain region was as follows: FP [female (91)/male (55)], STG [92/64], PHG [103/52], IFG [81/51], CBE [47/32], TCX [49/31], DLPFC [215/79], ACC [122/49], PCC [101/50] (Figure 3A). We identified a set of RNA editing events that were significantly associated with sex‐specific differences in AD brain, showing high heterogeneity across the nine brain regions (Tables S18–S26 and Figures S11–S19). Notably, the PHG exhibited the highest number of sex‐specific genes (158) in AD affected by RNA editing events, followed by DLPFC (150), IFG (91), and CBE (63) (Figure 3A).

Significantly distinguished sex‐specific AD target genes that we identified include *PLCXD1*, *LSAMP*, *KCNIP4*, *RBFOX1*, *FRMD5*, *LSAMP*, *LRRTM4*, *CSMD1*, *FRMD4A*, *CTNNA3*, *LRRTM3*, *ASMTL*, *EIF4G3*, *TNRC6A*, and *ANKRD30BL* (Figure 3A). Briefly, phosphatidylinositol specific phospholipase C X domain containing 1 (*PLCXD1*) is a complex locus present on X (Xp22.33) chromosomes mainly associated with the innate immune response.^98^ We observed that RNA editing in *PLCXD1* regions was significantly (*p* ≤ 5×10^3^) associated with AD in females across multiple brain regions (FP, STG, DLPFC, ACC, TCX). Limbic system associated membrane protein (LSAMP, 3q13.31) is an immunoglobulin neural cell adhesion molecule important in neuronal growth and axon guidance.^99^ Here, we observed that coedited regions on *LSAMP* [chr3:116003779‐116003815 (*p* = 0.00339), chr3:116244948‐116244987(*p* = 0.0049)] were negatively associated with AD in females in two brain regions (FP, ACC*). LSAMP* is also implicated in regulation of emotional and neuropsychiatric functions.^100^, ^101^ Likewise, two edited regions (chr15:44026070‐44026116 (*p* = 0.00358) and chr15:44122711‐44122760 (*p* = 0.00272)) of the *FRMD5* gene were correlated with male AD in FP and PHG, respectively. Two edited regions [chr10:67070607‐67070623 (*p* = 0.0013) and chr10:67005889‐67005928 (*p* = 0.0031)] from *LRRTM3* were also identified in PHG as associated with AD in males. Furthermore, RNA editing in the *ASMTL* (acetylserotonin O‐methyltransferase like) gene, located in the pseudoautosomal region 1 (PAR1) of X chromosome in the DLPFC, ACC, PCC, and CBE brain regions, correlated with AD in females. Notably, ASMTL‐AS1 is a long non‐coding RNA that was recently indicated as a potential biomarker of AD.^102^

Similarly, we also found that co‐edited regions in *LRRTM4* from distinct brain regions (FP [chr2:76864535‐76864574 (*p* = 0.00386)], IFG [chr2:77300025‐77300031 (*p* = 0.00344)], and PCC [chr2:77127886‐77127900 (*p* = 0.00187), chr2:77486011‐77486028 (*p* = 0.00377)]) were linked to female AD. In addition, RNA editing in *FRMD4A* at chromosome 10 in STG (*p* = 0.00353), PHG (*p* = 0.0035), and IFG (*p* = 0.00268) was positively correlated with AD. Likewise, *CSMD1* RNA editing in three brain regions (STG [chr8:3836005‐3836079 (*p* = 0.0044)], PHG [chr8:4693770‐4693780 (*p* = 0.0008)], and IFG [chr8:4317586‐4317617 (*p* = 0.00225)]) was significantly corelated with AD in females. Correspondingly, trinucleotide repeat‐containing gene 6A protein (TNRC6A) mediates miRNA‐induced gene silencing involved in RNA degradation pathways and is also implicated in epilepsy along with *RAPGEF2*.^103^, ^104^ Similarly, ankyrin repeat domain 30B like (ANKRD30BL, 2q21.2), catenin alpha 3 (CTNNA3, 10q21.3), and eukaryotic translation initiation factor 4 gamma 3 (EIF4G3, 1p36.12), all previously implicated in neurodegenerative diseases,^105^ were also found to undergo sex‐specific RNA editing. Shared pool of sex‐specific RNA edited genes from different brain regions is shown in the Venn diagram (Figure S20A, C, and E). The differential pattern of these RNA editing events from the nine brain regions is depicted using volcano plots and mirrored Manhattan plot to show the pattern of sex‐specific and APOE4‐specific genome‐wide RNA editing in AD (Figures S11–S19).

### 3.4 *APOE*‐specific differential RNA editing in the human AD brains

We further examined differences in RNA editing between AD subjects with *APOE4* (4/4, 3/4) versus non‐APOE4 genotypes from individual brain regions. The group‐wise number of samples from each brain region was as follows: FP (non‐APOE4 (82)/APOE4 (64)), STG (87/69), PHG (89/66), IFG (77/55), CBE (38/41), TCX (38/42), DLPFC (173/121), ACC (97/74), and PCC (93/58) (Figure 3B). Here, we discovered RNA editing clusters associated with *APOE4* in AD from all nine brain regions (Tables S27–S35). We also detected a mixed editing distribution in separate brain regions, which was characterized for affected target genes at a significant *p*‐value ≤ 0.005 (Figure 3B). Many *APOE4*‐specific RNA editing associated genes were identified from FP (141), IFG (133), ACC (123), DLPFC (111), CBE (79), PHG (65), STG (58), PCC (52), and TCX (16), as shown in Figure 3B. The *APOE4*‐specific genome‐wide editing patterns from distinct brain regions is depicted in Figures S11–S19. We found very few *APOE4*‐specific differentially edited genes shared across multiple brain regions (Figure S20B, D, and F).

The identified perturbed RNA edited target genes include *RBFOX1*, *TNRC6A*, *KCNIP4*, *RORA*, *PITPNC1*, *WAC*, *DPP6*, *FRRS1L*, *LRP1B*, *KIF13A*, *HSPA12A*, *CADM2*, *FRMD4A*, *MACF1*, and *SORBS1*. Interestingly, retinoic acid receptor (RAR)‐related orphan receptor A (RORA, 15q22.2) plays a role in metabolism and inflammation and has been shown to protect neuronal cells^106^, ^107^ and involved in various neurodegenerative diseases.^106^, ^107^, ^108^ Similarly, phosphatidylinositol transfer protein cytoplasmic 1 (PITPNC1, 17q24.2) is related to lipid metabolism, neuronal activity, and excitability.^109^ Furthermore, LDL receptor related protein 1B (LRP1B, 2q22.2) is a member of low‐density lipoprotein (LDL) receptor family and a key regulator of tau spread and aggregation in dementia.^110^, ^111^ The cell adhesion molecule 2 (CADM2, 3p12.1) plays critical roles in synaptic organization, glutamatergic signaling, and neuronal adhesion in dementia and psychiatric disorders,^112^, ^113^, ^114^ and sorbin and SH3 Domain Containing 1 (SORBS1, 10q24.1) plays an important role in insulin signaling and glucose homeostasis and has been linked to cognitive impairment.^115^ Lastly, the heat shock protein A12A (HSPA12A, 10q25.3) is a member of the HSP70 family that regulates protein misfolding and plays a vital role in multiple neuropsychiatric conditions, including AD, major depressive disorder, bipolar disorder, and schizophrenia.^116^, ^117^

Correspondingly, kinesin family member 13A (*KIF13A*, 6p22.3) is a component of axonal transport implicated in developmental and neurodegenerative diseases.^118^ Ferric chelate reductase 1 like (*FRRS1L*, 9q31.3) interrupts synaptic functions associated with neurodevelopmental and cognitive abnormalities related to glutamatergic signaling,^119^, ^120^ and the WW domain containing adaptor with coiled‐coil (WAC, 10p12.1) protein plays an important role in brain development and has been linked to intellectual disability, autism spectrum disorder, and developmental disorders.^121^, ^122^, ^123^, ^124^ Dipeptidyl peptidase like 6 (DPP6, 7q36.2) influences neuronal hyperexcitability and synaptic growth and has been linked to amyotrophic lateral sclerosis (ALS) and developmental disorders,^125^, ^126^ while the microtubule actin cross‐linking factor 1 (MACF1, 1p34.3) protein [also known as actin crosslinking factor 7 (ACF7)] is essential for actin‐microtubule interaction and linked to neurodevelopmental and neurodegenerative diseases, including AD,^127^, ^128^ schizophrenia,^129^ PD,^130^ and bipolar disorder.^131^

### 3.5 Brain‐wide *cis‐*edQTLs

**FIGURE 4:**
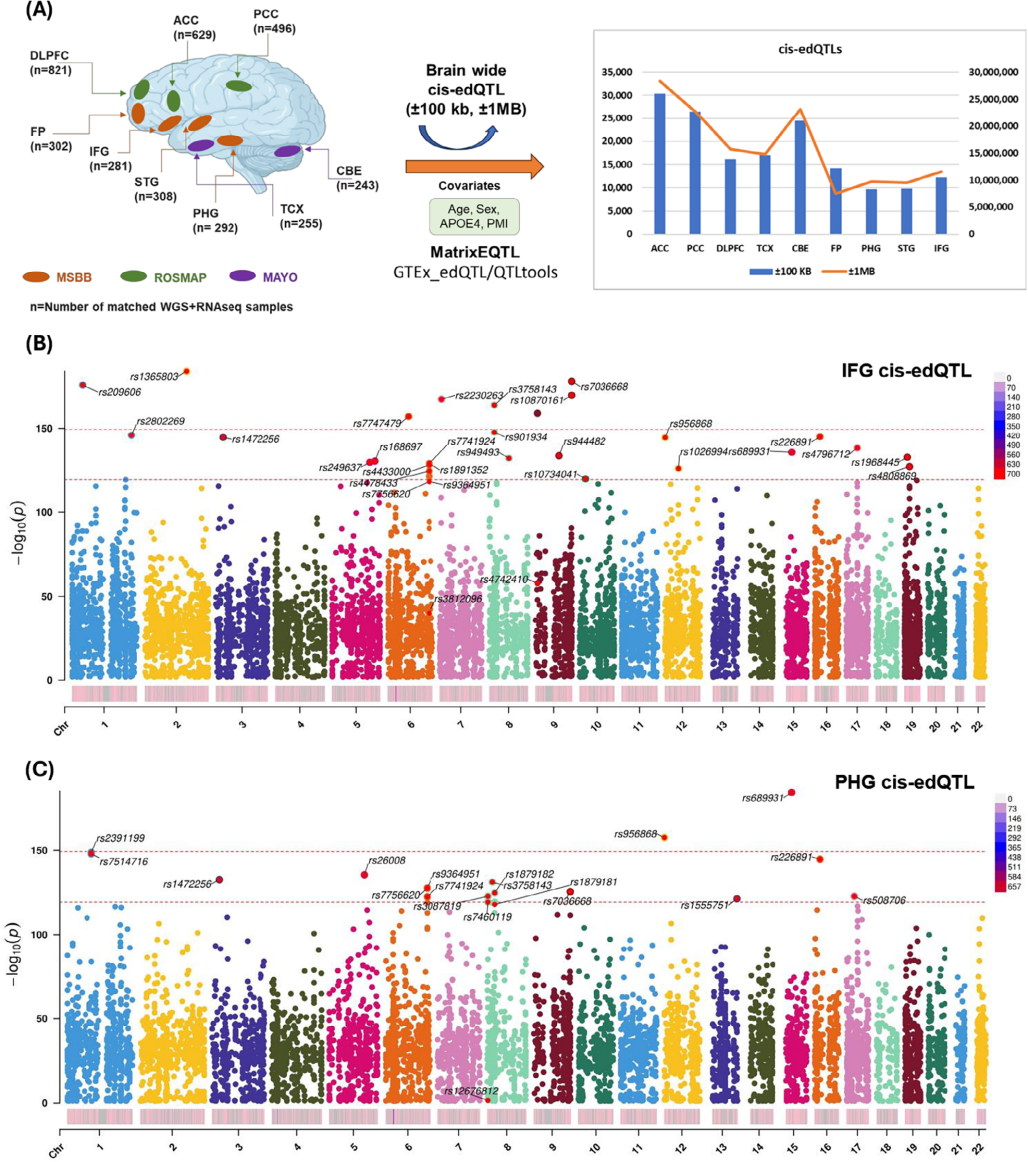
Brain‐wide *cis*‐regulatory variants (RNA editing quantitative trait loci [*cis*‐edQTLs]). (A) edQTL study overview showing number of samples from each brain region and covariates, Manhattan plot showing genome wide distribution of *cis*‐edQTLs from (B) IFG, (C) PHG brain tissues. IFG, inferior frontal gyrus; PHG, parahippocampal gyrus.

To elucidate the genetic variants that influence RNA editing at specific sites, we searched for SNPs associated with RNA editing levels with the goal of providing insights into underlying regulatory mechanisms, disease pathogenesis, and therapeutic targets. Specifically, we investigated genetic regulation of brain‐wide RNA editing sites in the nine brain regions for which we had access to matched WGS and RNAseq data (Table S1): FP (*n* = 302 samples), STG (*n* = 308), PHG (*n* = 292), IFG (*n* = 281), TCX (*n* = 255), CBE (*n* = 243), DLPFC (*n* = 821), ACC (*n* = 629), and PCC (*n* = 496) (Figure 4A).

Matched genotype data were utilized to detect SNPs with potential effect on RNA editing events in the brain. Separate RNA editing level matrices were prepared, filtered, and normalized, employing GTEx_edQTL/QTLtools package for each brain region. Furthermore, associated genetic variants (SNP genotype) with covarying age, sex, *APOE4* allele, and PMI were identified using a linear model implemented through the MatrixEQTL package for filtered editing data. To discover brain‐wide RNA editing events affected by genetic variations (*cis*‐edQTLs, SNP‐editing pairs), association was performed for the fixed windows of ± 100 kilobases (kb) and broader ± 1 megabase (mb) (Figure 4A).

In total, we identified 14,155; 9748; 9650; 12,236; 16,996; 24,449; 16,170; 30,386; and 26,475 *cis*‐edQTLs in the FP, STG, PHG, IFG, TCX, CBE, DLPFC, ACC, and PCC, respectively, at a genome‐wide FDR (FDR ≤ 0.05) within ± 100 kb distance window (Figures 4A and S21A). An overview of genome‐wide distribution of *cis*‐edQTLs is illustrated by Manhattan plots for IFG (Figure 4B) and PHG (Figure 4C). Subsequently, at ± 1 MB distance window, we obtained a large repertoire of *cis*‐edQTLs in nine brain regions (Figure S21A).

For *cis* regulating elements (CRE) of sites of RNA editing, we found from one‐to‐many and many‐to‐one relationships of RNA editing sites to SNPs. Notably, many edQTLs were highly correlated (low *p*‐values abundance), probably due to linkage disequilibrium (LD) as reported previously in schizophrenia.^34^ Genome‐wide distribution of the *cis*‐edQTLs was represented through Manhattan plots for individual brain regions from distinct biobanks (Figures S21–S23).

### 3.6 Discovery of likely causal genes in AD by co‐localizing with *cis‐*edQTLs

Next, we examined *cis*‐edQTL signatures to identify co‐localized signals with significant AD GWAS associations. Here, RNA editing was shown as an additional biological trait that might affect the GWAS variant loci and disease risk. We utilized the latest filtered GWAS summary statistics (FDR ≤ 0.05) from Bellenguez et al. (2022) and a brain‐region specific edQTLs set, which identified a set of 147 GWAS‐edQTL loci related to 48 target genes from multiple brain regions (Tables S36–S44).

**FIGURE 5:**
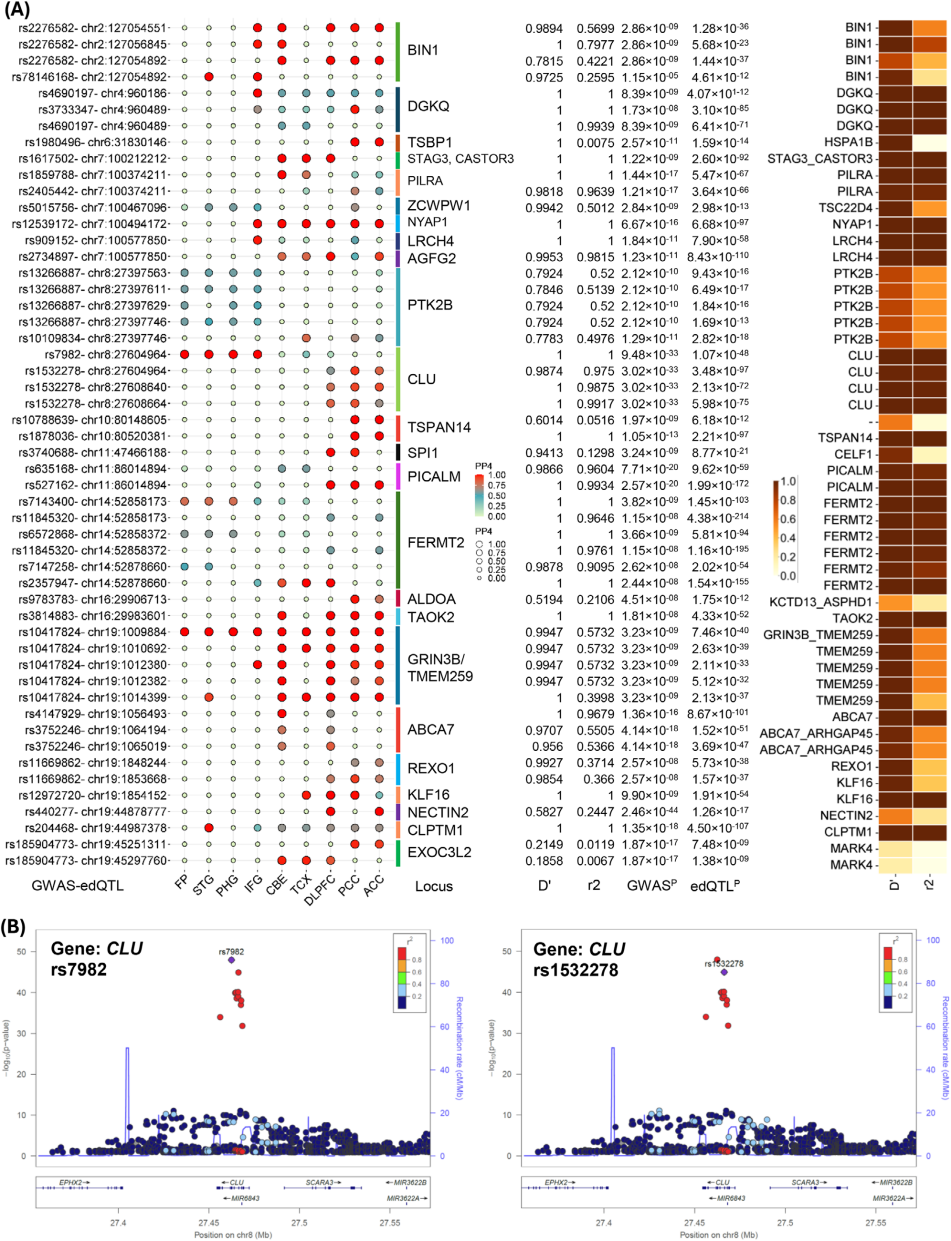
(A) Plot illustrating GWAS‐(*cis*) edQTLs colocalization (PP4 ≥ 0.50) across different brain tissues. Each row shows AD GWAS (Bellenguez et al. 2022) SNP and *cis*‐edQTLs pairs and each column indicates specific brain regions. (B) Regional association plot for the *CLU* locus with the top *cis*‐edQTL colocalized signals, that is, rs7982, rs1532278. Colors of each dot in LocusZoom plot indicate LD (*r* ^2^). edQTLs, RNA editing quantitative trait loci; GWAS, genome‐wide association studies.

In addition, the PP of colocalization between these association signatures (GWAS locus and edQTLs) employing coloc2 (Bayesian function) identified likely shared causal variants associated with RNA editing events contributing to AD pathology (Figure 5A). We thus identified key colocalized GWAS and *cis*‐edQTL (± 1 MB) genomic signals in 33 genes, including *CLU* (rs7982, rs1532278), *DGKQ* (rs4690197, rs3733347), *GRIN3B* (rs10417824, rs1058603), *BIN1* (rs2276582, rs3768863), *PICALM* (rs527162), and *NYAP1* (rs12539172). The bubble plot illustrates GWAS‐(*cis*) edQTLs colocalization (PP4 ≥ 0.50) along with locus and metainformation across different brain regions (Figure 5A). A comprehensive list of key biological functions and disorders linked to these genes is provided in Table S45. Briefly, the *CLU* (clusterin or apolipoprotein J) gene on chromosome 8 is a multifunctional glycoprotein transporter linked to β‐amyloid formation, lipid transport, inflammation, neuronal transmission, and chaperone activity.^132^, ^133^ It has been implicated in a number of diseases including cancer, inflammatory diseases, and neurodegenerative diseases like Huntington disease and AD. Distinct studies have suggested the association of *CLU* with the increased risk of AD and cognitive decline.^132^, ^134^, ^135^, ^136^, ^137^ Similarly, *BIN1* (bridging integrator 1) is a major genetic risk factor and considered to be the second most important AD risk gene.^24^, ^138^ It plays a vital role in tau pathology, synaptic plasticity, Aβ production, inflammation, calcium homeostasis, and neurotransmission.^138^, ^139^, ^140^, ^141^ Further, *PICALM* (phosphatidylinositol binding clathrin‐assembly protein) gene is another well‐known significant genetic susceptible locus associated with AD. It is a clathrin‐adaptor protein that can modulate Aβ production, transportation and clearance, one of the essential component or hallmark of AD pathology.^135^, ^142^

Correspondingly, *CLPTM1* (Cleft lip and palate transmembrane protein 1) gene encodes a multi‐pass transmembrane protein. It has been implicated to play a critical role in influencing synaptic strength through GABAA receptor regulation.^143^ It has also been indicated to be associated with AD using the integrated transcriptome‐wide association studies (TWAS).^144^, ^145^ *GRIN3B* (Glutamate receptor ionotropic NMDA subunit 3B) is one of the essential member genes that encodes a subunit of the N‐methyl‐D‐aspartate receptor (NMDA receptor), a heterotetrameric ion channel that plays a vital role in synaptic transmission, neuroplasticity, and memory functions.^146^ Abnormalities in NMDA receptors and set of genes including *GRIN3A*, *GRIN2B*, and *GRIN3B* were implicated in schizophrenia, epilepsy, and AD.^147^, ^148^, ^149^, ^150^, ^151^, ^152^, ^153^ *DGKQ* (diacylglycerol kinase theta) gene is known to modulate lipid metabolism and cell signal transduction. It has been linked to obesity related disorders and type 2 diabetes.^154^ It has also been associated with the PD^155^, ^156^ and AD.^24^ Additionally, the DGKs family of kinases were implicated in various roles involving neural and immune responses, cancer and neurological disorders, including autism, bipolar disorders, cognitive impairments, and intellectual disability.^157^ Likewise, *NYAP1* (neuronal tyrosine‐phosphorylated phosphoinositide‐3‐kinase adapter 1) locus encodes numerous prioritized significant AD GWAS genes, including *AGFG2*, *PILRA*, and *EPHB4*.^24^, ^25^, ^158^, ^159^ It has been involved in neuronal morphogenesis while regulating PI3K signaling pathway potentially impacting neuronal connectivity and synaptic plasticity, critical features of AD pathology.^160^, ^161^ Some prominent RNA editing variant signals colocalized with the significant AD‐associated GWAS loci are explained in the following section.

**FIGURE 6:**
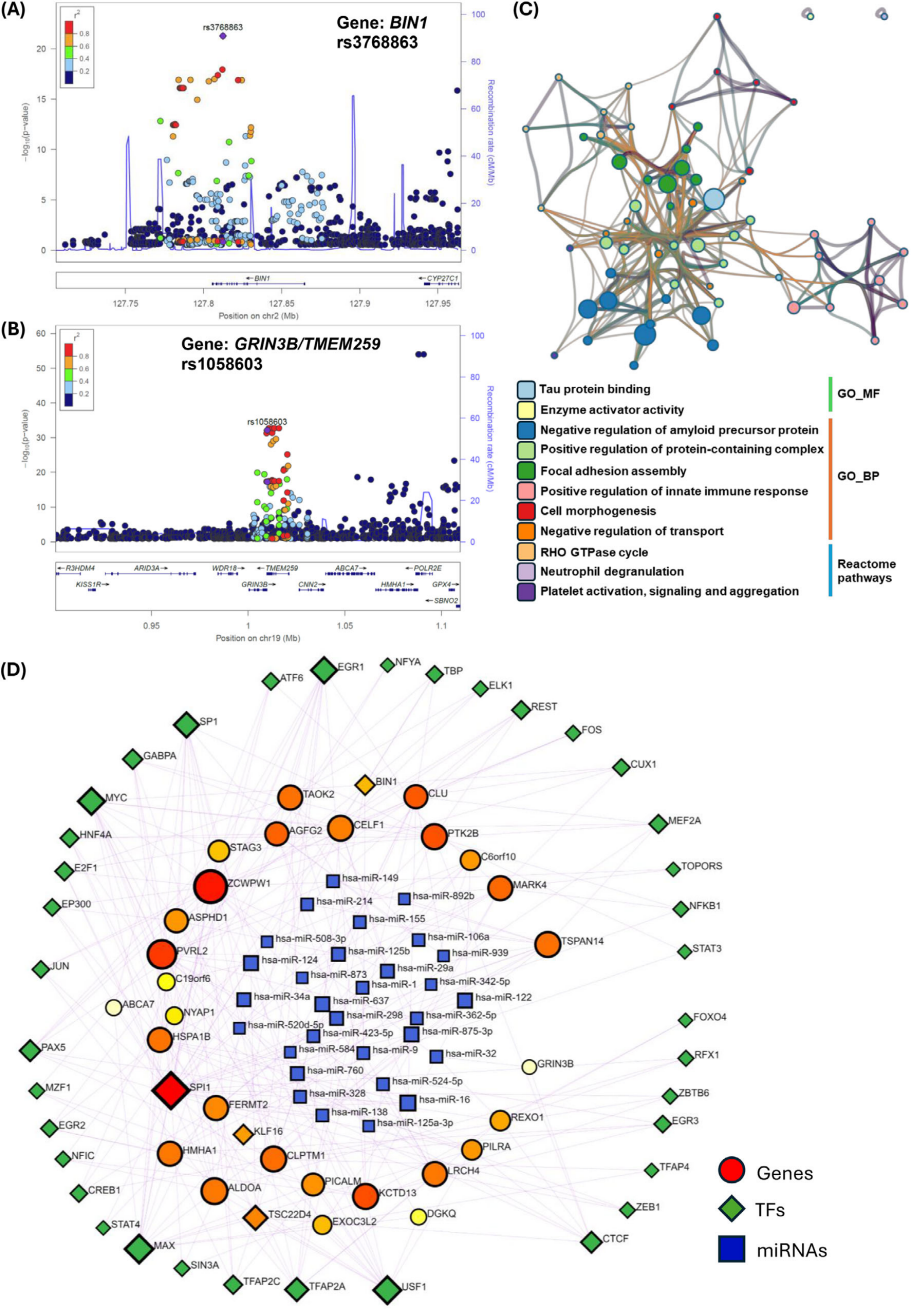
Regional association plot for (A) BIN1 (rs3768863, chr8), (B) GRIN3B/TMEM259 (rs1058603, chr19) with the top *cis*‐edQTL colocalized signals. Colors of each dot in LocusZoom plot indicate linkage disequilibrium (LD) (*r* ^2^). (C) Cytoscape cluster network representing functionally enriched pathways in key colocalized gene signals, (D) Gene coregulatory network (GRN) depicting TF‐Gene‐miRNAs relationship. Where, circle and orange color represent genes, diamond and green depict transcription factor (TFs), and rectangular blue color shows regulatory microRNAs (miRNAs). Node size displays number of coregulatory relationships. edQTLs, RNA editing quantitative trait loci.

Integrating molecular *cis*‐edQTLs and AD GWAS data revealed edQTL‐GWAS pairs in the LD block pertaining to well‐known AD‐targets, such as *CLU* and *BIN1*. This indicates the potential role of an epitranscriptomic mechanism influencing AD risk. Notably, we identified four colocalizations at the *CLU* locus (Figure 5A). SNPs rs7982 and rs1532278 are associated with the edQTLs within the *CLU* gene region with strong colocalization probability (PP4 ≥ 0.70) across distinct brain regions. The regional association LocusZoom plot depicts the *cis*‐edQTL colocalized signals (rs7982, rs1532278) for the *CLU* locus on chromosome 8 (Figure 5B). These colocalized SNPs are in high LD (*r* ^2^ ≥ 0.95 in the 1000 Genomes EUR population) with the AD GWAS SNPs (Figure 5B). Similarly, four colocalized pairs were recognized at the *BIN1* locus with high PP4 more than 0.99 pertaining to distinct brain tissues (Figure 5A, Tables S36–S44). Likewise, another LocusZoom association plot shows colocalized signals in *BIN1* (rs3768863) at chromosome 2 (Figure 6A). Correspondingly, multiple pairs of edQTL‐GWAS variants associated with *GRIN3B/TMEM259* were colocalized with a high probability score ≥ 0.99. The AD GWAS SNP (rs10417824) colocalized with the edQTL signal (rs1058603) with high significance and was held in common across all brain regions (Figure 5A, Tables S36–S44). A LocusZoom association plot depicts the colocalized edQTL variant (rs1058603) at the GRIN3B locus at chromosome 19 (Figure 6B).

Furthermore, patterns of shared colocalized signals across brain tissues highlight potential clues to AD or neurodegenerative disorder pathway perturbations, and functional enrichment analysis shows these colocalized genes are related to tau protein binding, amyloid‐β regulation, immune pathways, platelet activation and signaling, neutrophil degranulation, AD disease pathways, protein metabolism, and signal transduction (Figure 6C). Furthermore, the identified key colocalized genes (33) were used to deduce the gene regulatory network (GRN) to recognize relevant miRNAs and TFs (TF–Gene–miRNAs network) (Figure 6D).

## 4 DISCUSSION

RNA editing is at the forefront of epitranscriptomic modifications, associated with several functions from regulating RNA stability and alternative splicing to its role in neuronal dynamics and immune response. Studying RNA editing signals could pave the way for identifying potential biomarkers and therapeutic modalities in AD. In this study, we have comprehensively investigated genome‐wide landscape of RNA‐editing events from 4208 (1364 AD cases vs. 742 healthy controls) RNA‐seq samples across nine human brain regions from three large biobanks (MSBB, MAYO, ROSMAP), including sex‐specific and *APOE4*‐specific manner adjusting for age, PMI, sex, and *APOE4* status. Further, we examined brain‐wide *cis*‐regulatory variants (*cis*‐edQTLs) utilizing matched genotyping data from 3627 samples across all brain biobanks. Later, colocalization analysis has been performed to discover likely causal genes utilizing *cis*‐edQTLs and AD GWAS summary statistics. Overall, we emphasize 127 genes with significant RNA editing loci from the large repertoire shared across multiple brain regions. We also revealed 147 colocalized GWAS and *cis*‐edQTLs signatures in 48 likely causal genes. Our findings underscore the significance of RNA editing in AD, highlighting its role in neuronal and synaptic regulation. The identification of sex‐specific and *APOE4*‐related editing patterns further elucidates the complexity of AD pathophysiology.

Most of the associated RNA edited genes in AD from different brain regions that we identified were related to neuronal and synaptic regulation. These genes were found to encompass previously documented aberrantly edited genes, including *SYT11*, *KCNIP4*, *RBFOX1*, *GABRD*, and *LRRTM4* (Figure 2C), along with less studied gene such as *RALYL*, *NRG3*, *FRMD5*, *NRXN3*, *ATRNL1*, and *NLGN1*. *SYT11*, which regulates immune functions, neurotoxicity, and synaptic plasticity, has been reported in various neurological disorders, including AD, PD, and schizophrenia.^72^, ^74^, ^75^ *KCNIP4* has a major function of regulating potassium channels in neuronal activity and morphogenesis. Thus, our results show that RNA editing in the AD brain perturbs neuronal and synaptic regulation, although the functional implications of this myriad altered RNA editing events remain to be determined.

Importantly, we found an association with the AD of RNA editing of *RALYL*, which controls post‐transcriptional miRNA processing and mitochondrial metabolism‐related genes. It also regulates RNA splicing of immune and inflammatory genes. Similarly, our research revealed that RNA editing of *NRG3* in AD, a neuregulin family gene located in 10q23.1 and involved in synaptic transmission and repair of nervous system. Likewise, multiple cell surface and adhesion proteins (*NRXN3*, 14q24.3; *NLGN1*, 3q26.31; *CNTNAP2*, 7q35; *FRMD5*) that are essential components of nervous system development along with synaptic function and transmission were also disrupted.

Moreover, we identified sex‐specific RNA editing differences in AD. For example, RNA editing in *PLCXD1* (Xp22.33) and *ASMTL* located in the PAR1 region were significantly associated with AD in females across different brain regions. *PLCXD1* is related to innate immune response and *ASMTL* is a critical component of neuronal growth and axon guidance. These genes have multiple transcript variants, which might be influenced through RNA editing in ways that could perturb their function. Notably, ASMTL‐AS1 was recently reported as a potential biomarker for AD.^102^

A large repertoire of brain‐wide *cis*‐edQTLs was detected as being specific to individual brain regions (Figure 4). Distribution of these edQTLs was highly diverse between different brain regions (Figure 4A), as ACC, PCC, and CBE exhibited the highest number of *cis*‐edQTL editing pairs and PHG the least. These RNA editing sites demonstrate association with one or more *cis*‐regulatory variants, and we found strong correlation between editing sites (low FDR) with the respective *cis*‐regulatory signals. This is probably due to the contribution of LD. However, to explore the likely causal relevance, GWAS‐edQTL co‐localization was performed for each brain region using the most recent AD GWAS‐summary statistics (FDR ≤ 0.05) from Bellenguez et al. 2022.

Overall, AD GWAS loci colocalized with *cis*‐edQTLs that were shared across different brain regions, including 33 genes of which many have been well‐documented in AD (i.e., *CLU*, *BIN*, *TSPAN14*, *PICALM*, and *CLPTM1*) (Figure 5A). This approach also incorporated edSNPs and editing sites from other potential genes, such as *NYAP1, DGKQ*, *ACE*, *FERMT2*, *GRIN3B*, and *AGFG2*. These are mainly linked with amyloid regulation, metabolism, neutrophil degranulation, and immune system function in AD (Figure 6C), indicating that RNA editing might facilitate key regulation comprising immune metabolism as well as amyloid mediated AD.

In conclusion, our comprehensive analysis reveals the intricate relationship between RNA editing and AD. Although aberrations in many of these genes have previously been linked to AD, we do not know whether all these RNA editing events are pathological, or whether some might be compensatory to help preserve brain function in AD. The large resource of transcriptomic and genotyping data has been explored to discover brain‐region specific events and a sizable landscape of RNA editing signals is presented in this study. These expanded relationships could be investigated further to determine their direct roles in AD. Predominantly, we have found key contributions in multiple aspects of AD pathophysiology, including synaptic regulation, β‐amyloid formation, neuronal toxicity, lipoprotein metabolism, and immune inflammatory mechanisms.

We acknowledge potential limitations of this study. Although we took multiple precautions toward harmonizing and analyzing the RNA‐seq data and WGS data, it is still challenging to achieve clinical ad genomic data harmonization. We utilized the curated and harmonized clinical data specific to each brain region available through an established RNA harmonization study and found reprises for some samples. This includes WGS data from the same individual in which brain tissue was analyzed as well as blood, with priority given to the brain tissue. Further, we lack data from more key AD brain regions including hippocampus and entorhinal cortex, which is not included in the present work. Additionally, the current scope of the RNA editing landscape is focused on the base substitutions and did not include the insertion and deletion in the current analysis. This study also does not focus deeply on RNA editing in non‐coding regions and it will be interesting to explore this in future studies in association with miRNA biogenesis and chromatin biology. Furthermore, cell‐specific RNA editing events could be investigated in future studies to interpret in depth biological and functional consequences of these events in a cell‐specific manner in AD. Also, incorporating long‐read sequencing data could help generate a more curated global RNA editing landscape. Additionally, we anticipate integrating multi‐omics data (such as ATAC‐seq and ChIP‐Seq) in the future to elucidate regulatory relationships of specific RNA editing sites. Nevertheless, functional validations will be required to translate discoveries as RNA editing‐based biomarkers or therapeutics.

## AUTHOR CONTRIBUTIONS

Feixiong Cheng conceived the study. Amit Kumar Gupta performed all genetic and genomic data analyses and experiments. William Martin help data analysis and computing. Andrew A. Pieper, Yinsheng Wang, and Andrew J. Saykin interpreted the data analysis. Amit Kumar Gupta and Feixiong Cheng drafted the manuscript. Amit Kumar Gupta, Feixiong Cheng, and Andrew A. Pieper critically revised the manuscript. All authors gave final approval of the manuscript.

## CONFLICT OF INTEREST STATEMENT

The authors have no competing interests. Author disclosures are available in the supporting information.

## Supporting information



Supporting Information

Supporting Information

Supporting Information
