# Supplementary material for: Comprehensive characterization of the RNA editing landscape in the human aging brains with Alzheimer's disease
Source: Alzheimers Dement. 2025 Jul 9;21(7):e70452. doi: 10.1002/alz.70452 (PMC12238832; doi:10.1002/alz.70452)
Supplement: Supplementary file 2 — Supporting Information [file ALZ-21-e70452-s003.pdf]

Supplementary Figures

Figure S1. Overview of the study workflow and design

Figure S2. Genomic spreading of RNA editing sites in different brain tissues (A) PHG, (B) IFG, (C) STG, (D) FP from MSBB cohort.

Figure S3. Genomic spreading of RNA editing sites in different brain tissues (A) TCX, (B) CBE from MAYO cohort.

Figure S4. Genomic spreading of RNA editing sites in different brain tissues (A) DLPFC, (B) ACC, (C) PCC from ROSMAP cohort.

Figure S5. Manhattan plots depicting genome-wide distribution of RNAedits from distinct tissues from MSBB, (A) PHG, (B) IFG, (C) STG, (D) FP, (E) number of genes at different significant threshold.

Figure S6. Manhattan plots depicting genome-wide distribution of RNAedits from distinct tissues from MAYO, (A) TCX, (B) CBE, (C) number of genes at different significant threshold.

Figure S7. Manhattan plots depicting genome-wide distribution of RNAedits from distinct tissues from ROSMAP, (A) DLPFC, (B) ACC, (C) PCC, (D) number of genes at different significant threshold.

Figure S8. Functional enrichment of RNA edited genes from four brain regions from MSBB cohort (A) PHG, (B) IFG, (C) STG, (D) FP.

Figure S9. Functional enrichment of RNA edited genes from CBE brain region from MAYO cohort.

Figure S10. Functional enrichment of RNA edited genes from two brain regions from ROSMAP cohort (A) DLPFC, (B) PCC.

Figure S11. Differential pattern of RNA editing events from PHG brain region, volcano plots showing (A) AD apoe4 vs AD nonapoe4, (B) AD female vs AD male, (C) mirrored Manhattan plot showing genome-wide distribution from both groups.

Figure S12. Differential pattern of RNA editing events from IFG brain region, volcano plots showing (A) AD apoe4 vs AD nonapoe4, (B) AD female vs AD male, (C) mirrored Manhattan plot showing genome-wide distribution from both groups.

Figure S13. Differential pattern of RNA editing events from STG brain region, volcano plots showing (A) AD apoe4 vs AD nonapoe4, (B) AD female vs AD male, (C) mirrored Manhattan plot showing genome-wide distribution from both groups.

Figure S14. Differential pattern of RNA editing events from FP brain region, volcano plots showing (A) AD apoe4 vs AD nonapoe4, (B) AD female vs AD male, (C) mirrored Manhattan plot showing genome-wide distribution from both groups.

Figure S15. Differential pattern of RNA editing events from TCX brain region, volcano plots showing (A) AD apoe4 vs AD nonapoe4, (B) AD female vs AD male, (C) mirrored Manhattan plot showing genome-wide distribution from both groups.

Figure S16. Differential pattern of RNA editing events from CBE brain region, volcano plots showing (A) AD apoe4 vs AD nonapoe4, (B) AD female vs AD male, (C) mirrored Manhattan plot showing genome-wide distribution from both groups.

Figure S17. Differential pattern of RNA editing events from DLPFC brain region, volcano plots showing (A) AD apoe4 vs AD nonapoe4, (B) AD female vs AD male, (C) mirrored Manhattan plot showing genome-wide distribution from both groups.

Figure S18. Differential pattern of RNA editing events from ACC brain region, volcano plots showing (A) AD apoe4 vs AD nonapoe4, (B) AD female vs AD male, (C) mirrored Manhattan plot showing genome-wide distribution from both groups.

Figure S19. Differential pattern of RNA editing events from PCC brain region, volcano plots showing (A) AD apoe4 vs AD nonapoe4, (B) AD female vs AD male, (C) mirrored Manhattan plot showing genome-wide distribution from both groups.

Figure S20. Sex-specific (A, C, E) and APOE4-specifc (B, D, F) RNA edited genes shared among multiple brain regions from three biobanks (A, B) MSBB, (C, D) MAYO, (E, F) ROSMAP.

Figure S21. (A) Illustrating number of samples and cis-edQTLs from the nine brain regions within ±100 KB and ±1 MB distance window. Genome-wide distribution of the cis-edQTLs pertaining to tissues from MSBB biobank (B) STG, (C) FP.

Figure S22. Genome-wide distribution of the cis-edQTLs from individual tissues, (A) TCX, (B) CBE from MAYO biobank.

Figure S23. Genome-wide distribution of the cis-edQTLs from distinct tissues, (A) DLPFC, (B) ACC, (C) PCC from ROSMAP biobank.

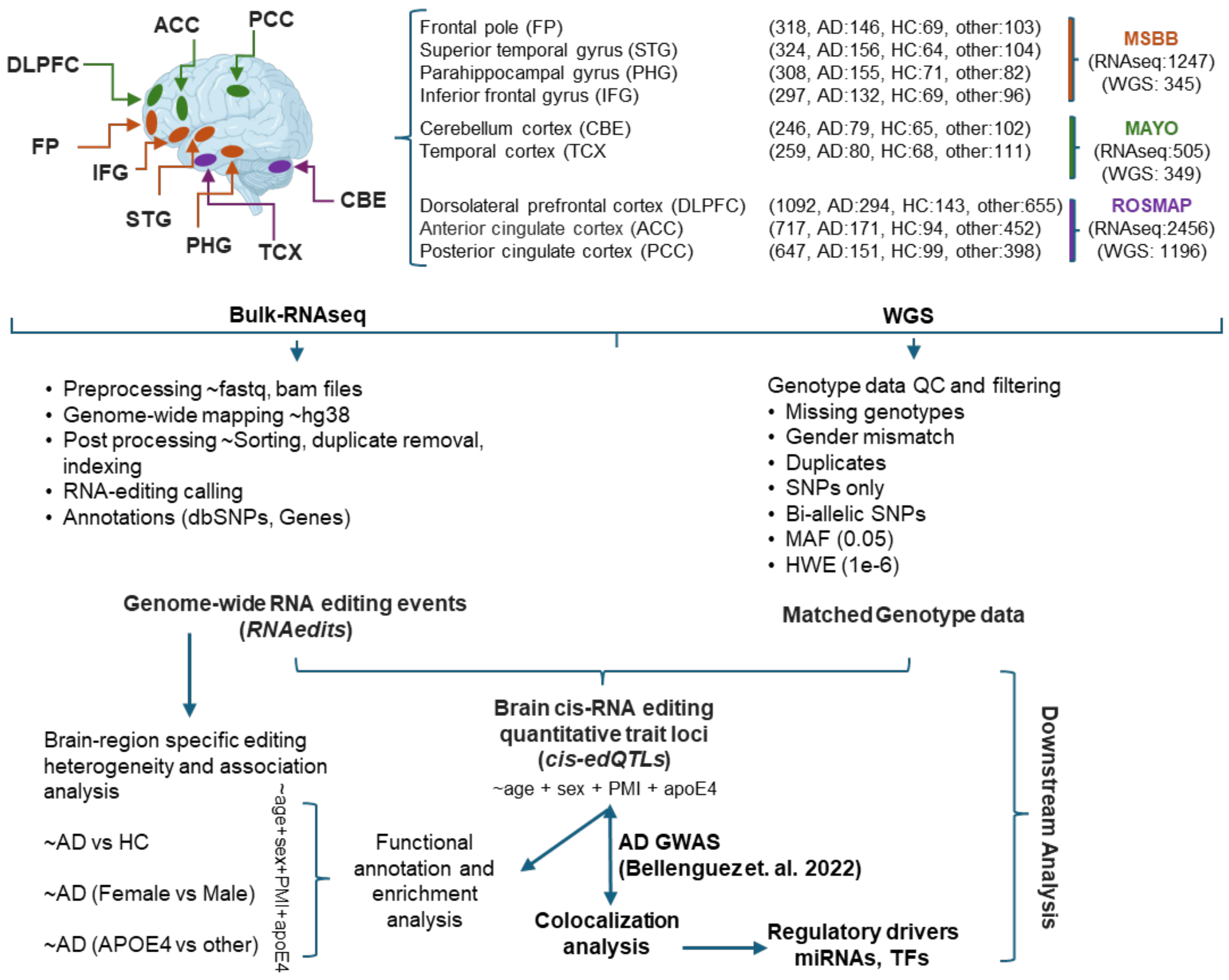

Figure S1. Overview of the study workflow and design.

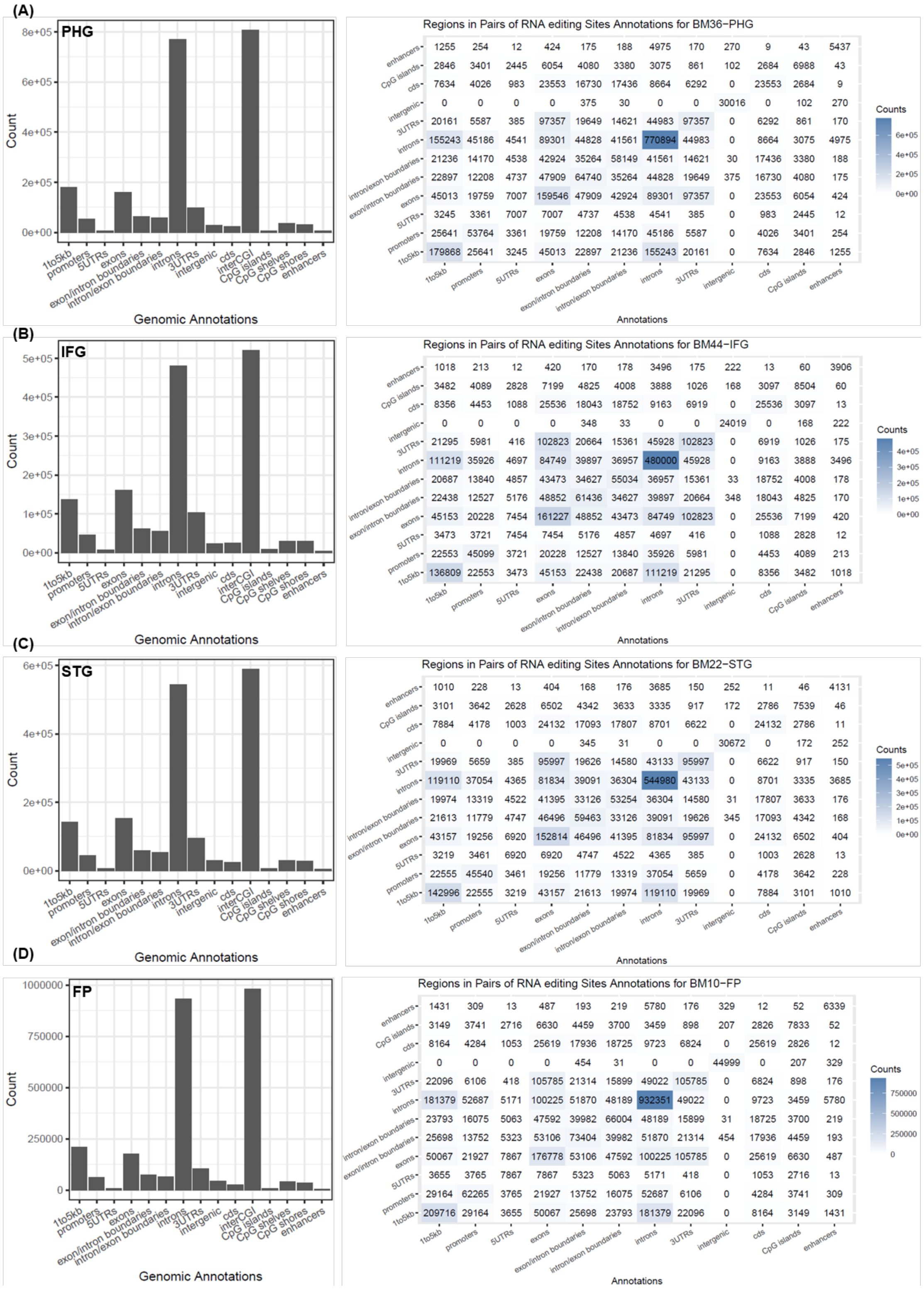

Figure S2. Genomic spreading of RNA editing sites in different brain tissues (A) PHG, (B) IFG, (C) STG, (D) FP from MSBB cohort.

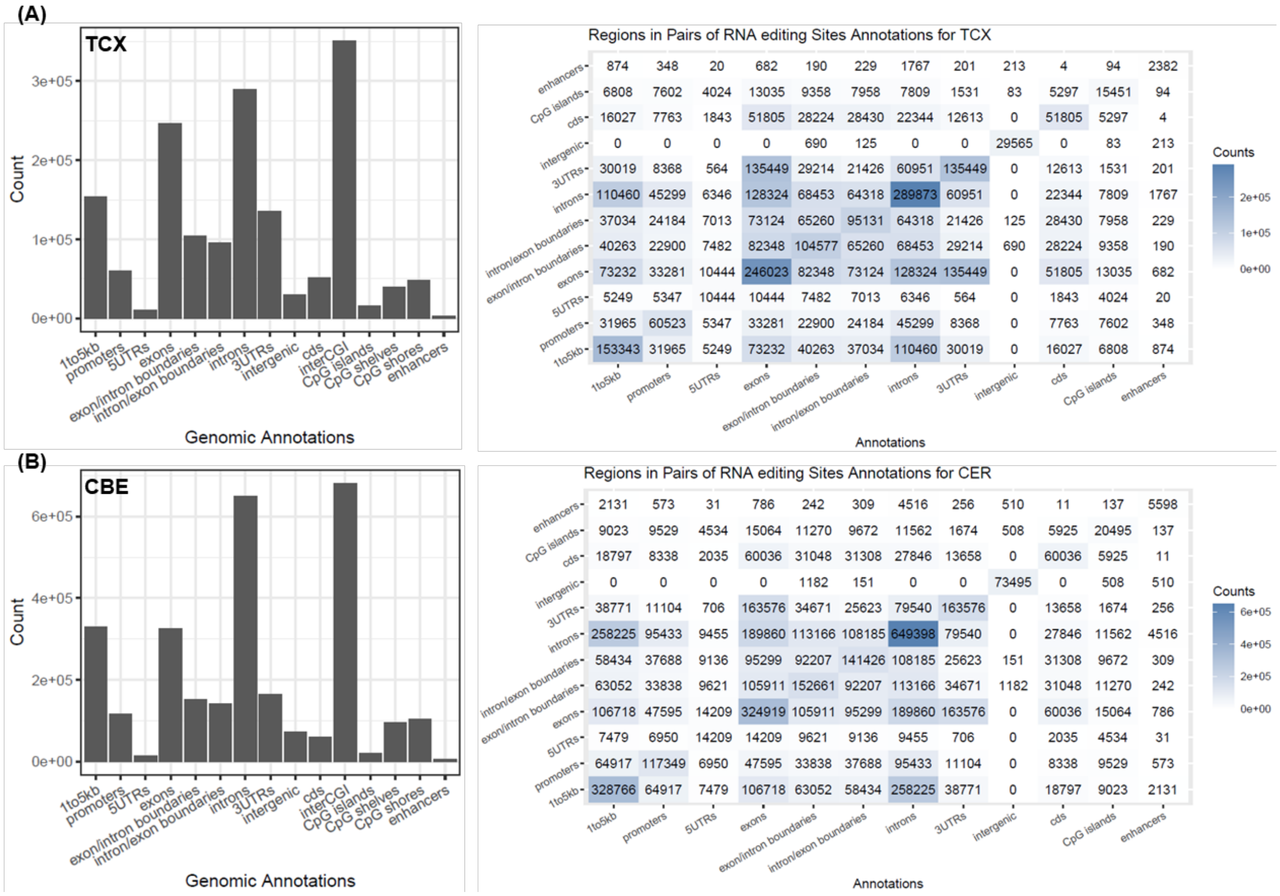

Figure S3. Genomic spreading of RNA editing sites in different brain tissues (A) TCX, (B) CBE from MAYO cohort.

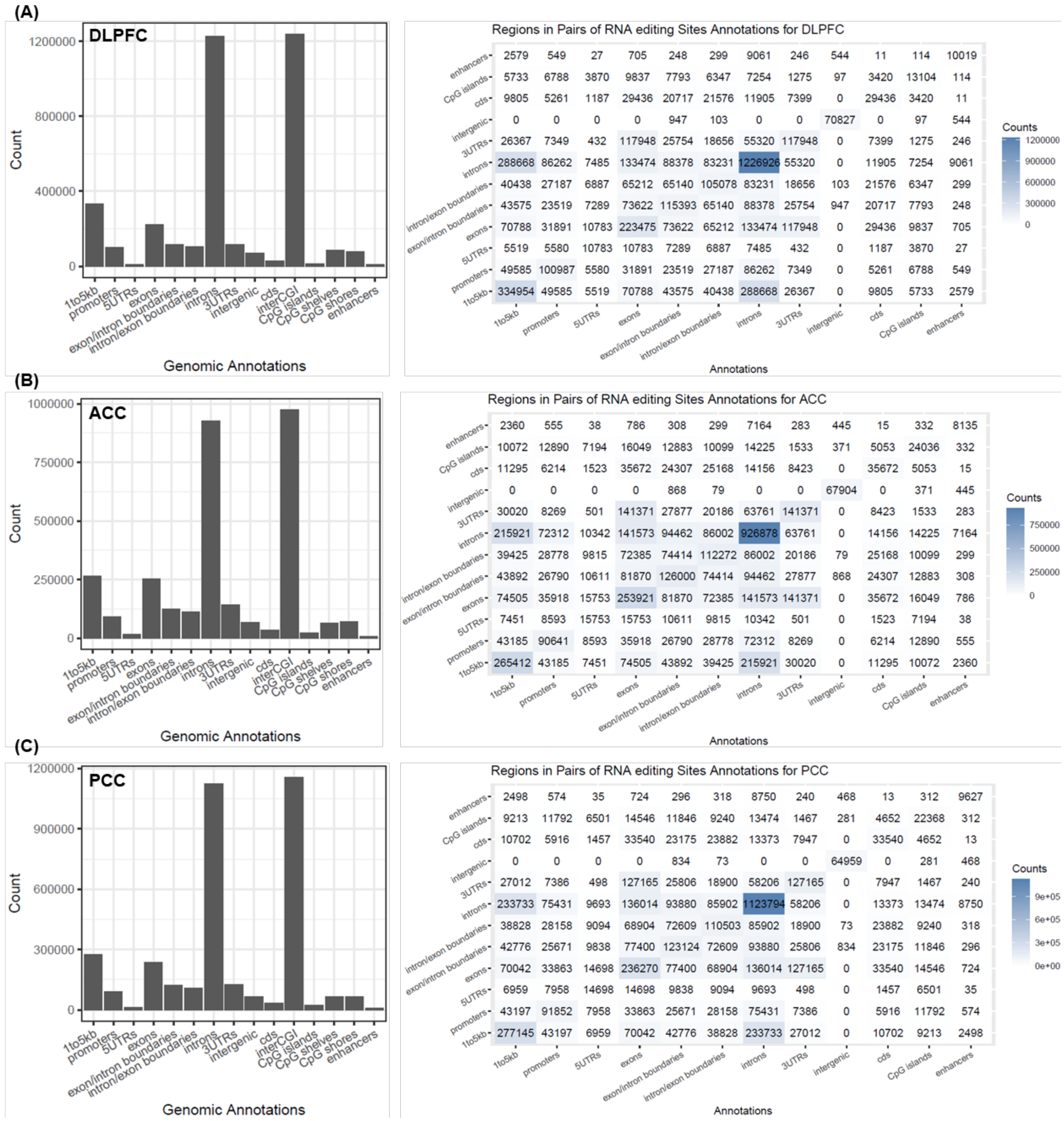

Figure S4. Genomic spreading of RNA editing sites in different brain tissues (A) DLPFC, (B) ACC, (C) PCC from ROSMAP cohort.

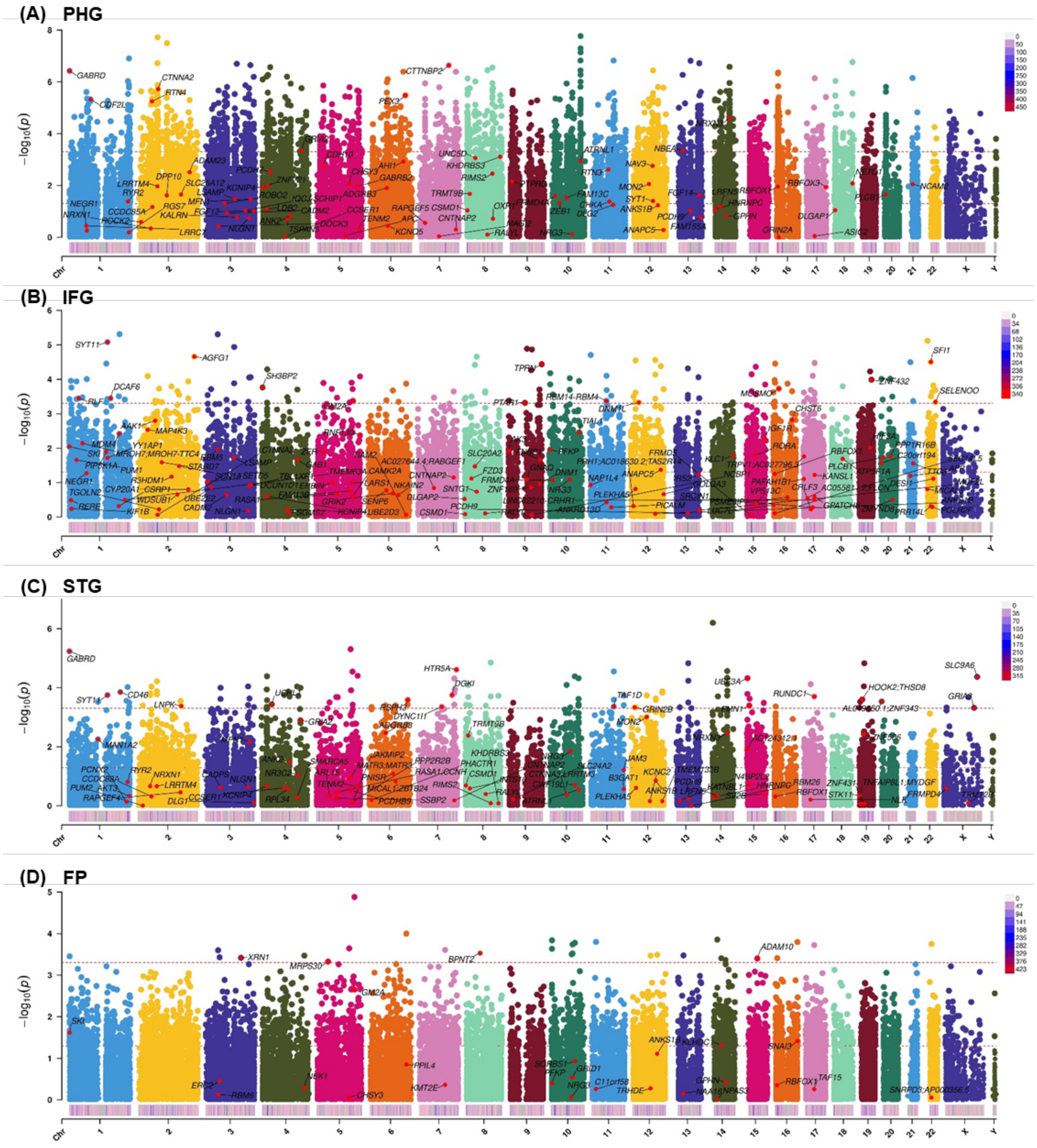

(E)

P-value	Ngenes			
	PHG	IFG	STG	FP
0.05	2537	2260	1478	2019
0.005	**1220**	**618**	**361**	**295**
0.0005	560	116	86	27
0.00005	226	22	17	
0.000005	82			

Figure S5. Manhattan plots depicting genome-wide distribution of RNAedits from distinct tissues from MSBB, (A) PHG, (B) IFG, (C) STG, (D) FP, (E) number of genes at different significant threshold.

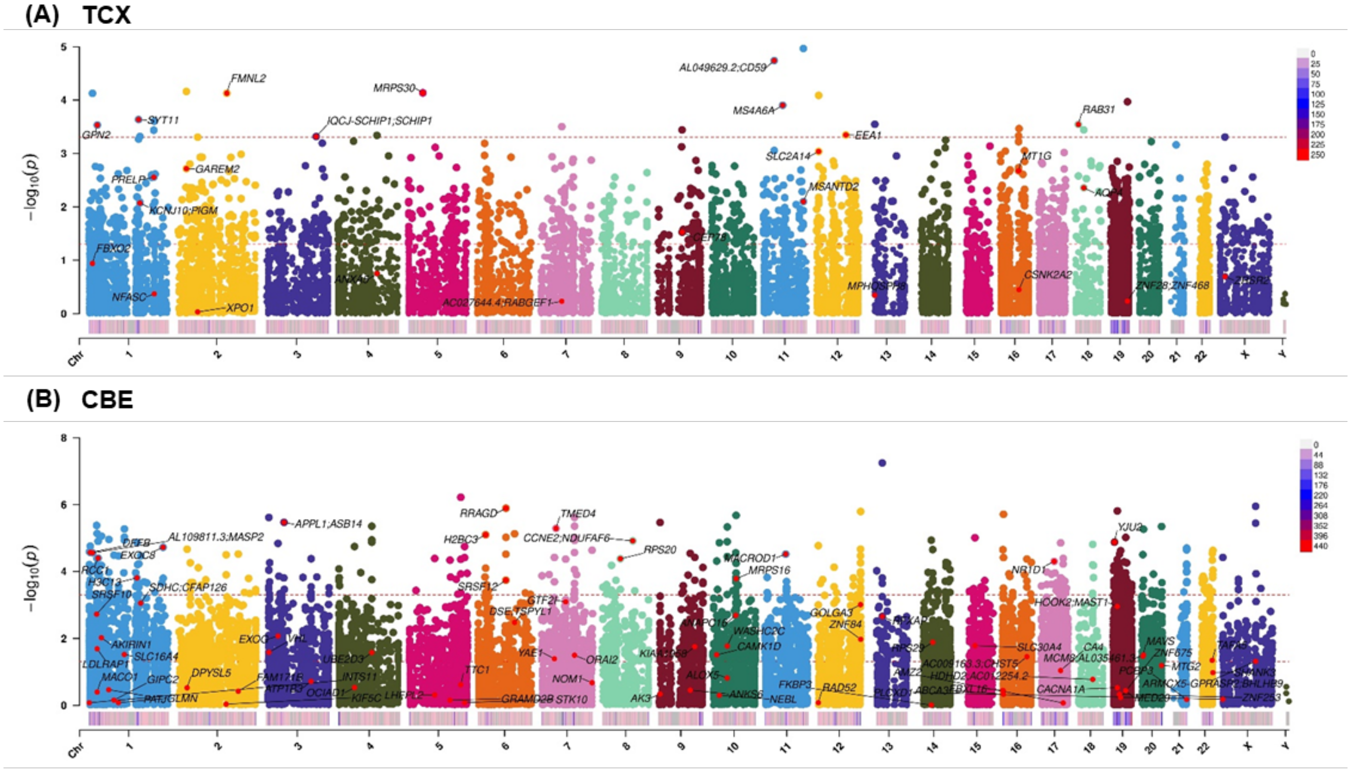

(C)

P-value	Ngenes	
	TCX	CBE
0.05	1308	3070
0.005	**209**	**981**
0.0005	26	262
0.00005		77

Figure S6. Manhattan plots depicting genome-wide distribution of RNAedits from distinct tissues from MAYO, (A) TCX, (B) CBE, (C) number of genes at different significant threshold.

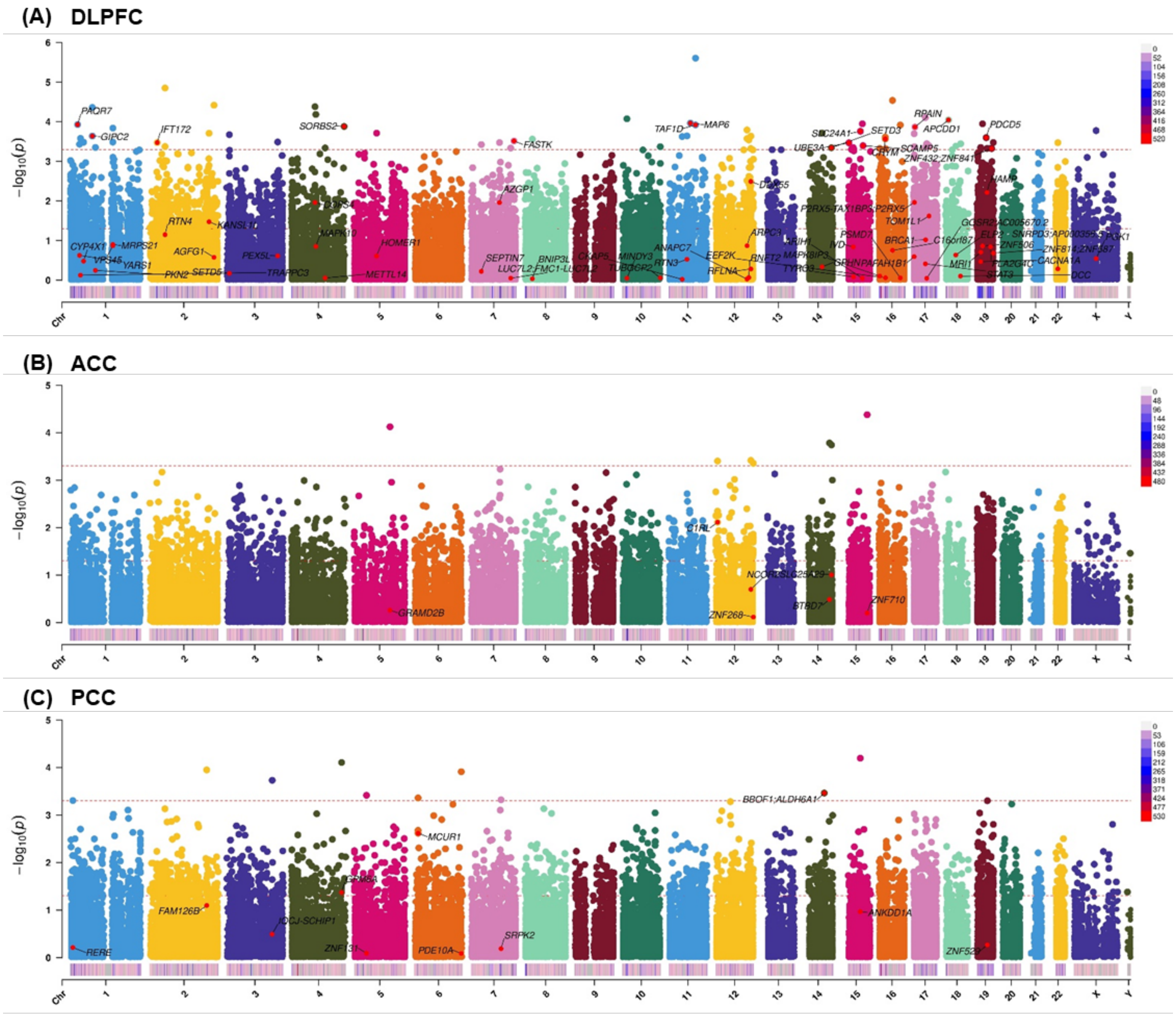

(D)

P-value	Ngenes		
	DLPFC	ACC	PCC
0.05	2621	1414	1560
0.005	**517**	**133**	**133**
0.0005	68	07	11

Figure S7. Manhattan plots depicting genome-wide distribution of RNAedits from distinct tissues from ROSMAP, (A) DLPFC, (B) ACC, (C) PCC, (D) number of genes at different significant threshold.

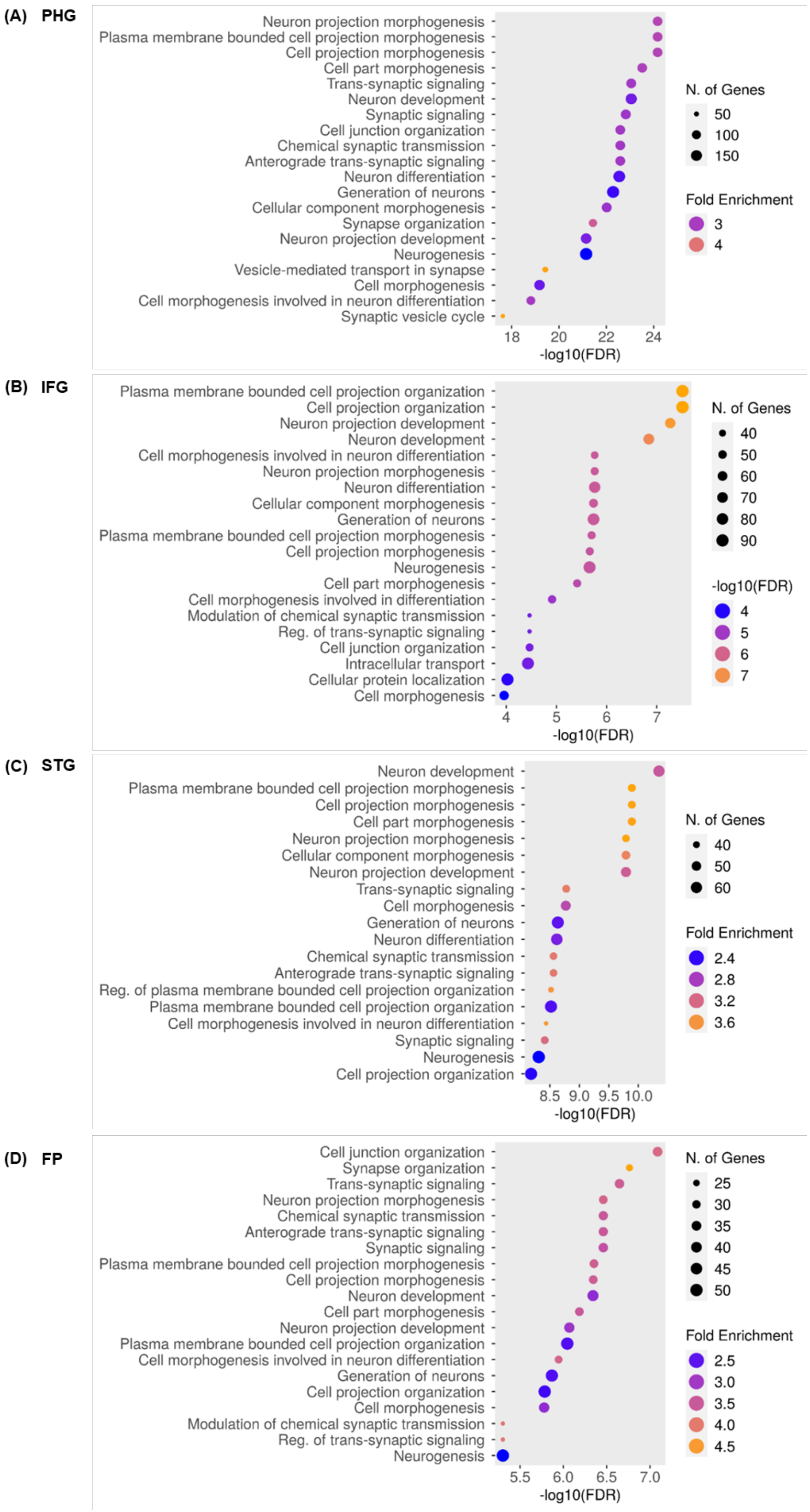

Figure S8. Functional enrichment of RNA edited genes from four brain regions from MSBB cohort (A) PHG, (B) IFG, (C) STG, (D) FP.

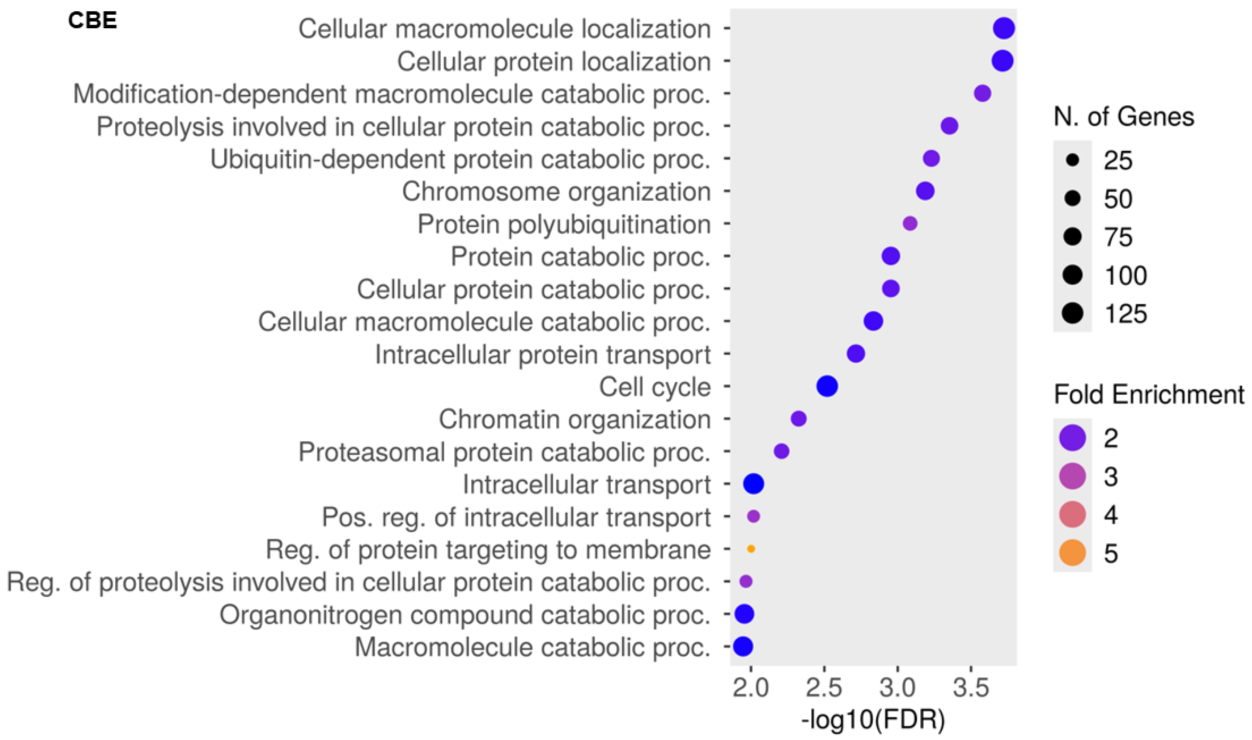

Figure S9. Functional enrichment of RNA edited genes from CBE brain region from MAYO cohort.

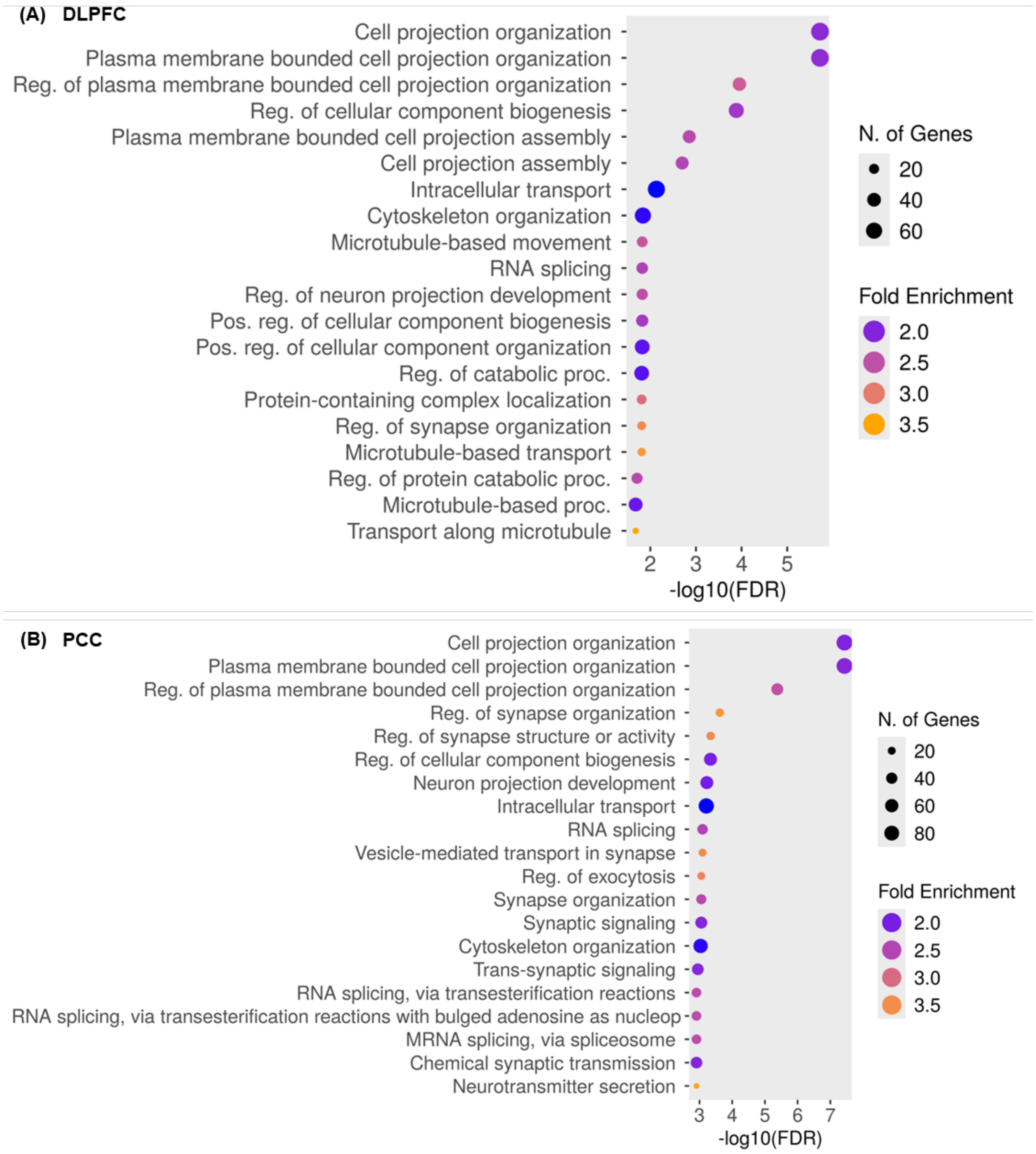

Figure S10. Functional enrichment of RNA edited genes from two brain regions from ROSMAP cohort (A) DLPFC, (B) PCC.

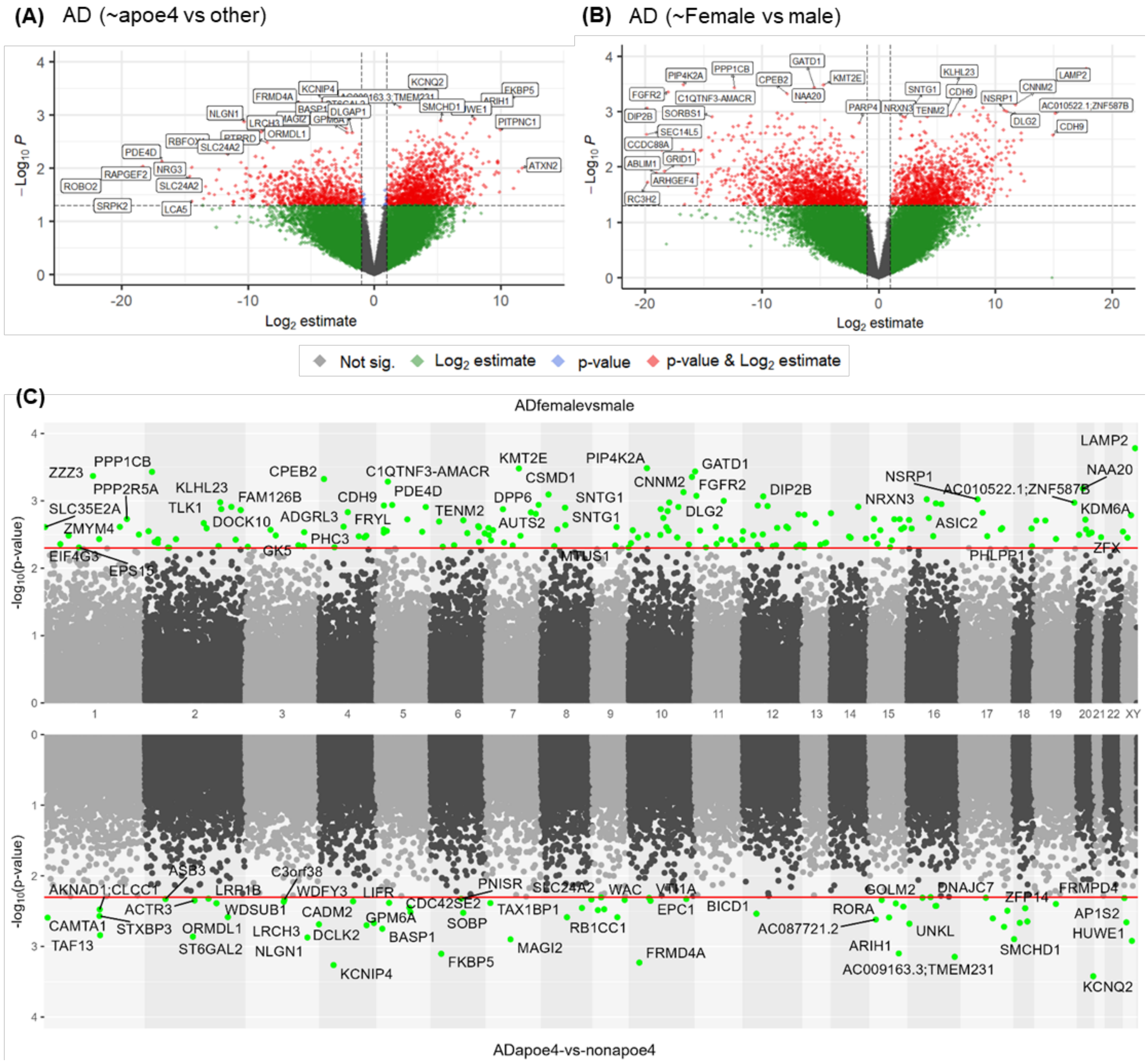

Figure S11. Differential pattern of RNA editing events from PHG brain region, volcano plots showing (A) AD apoe4 vs AD nonapoe4, (B) AD female vs AD male, (C) mirrored Manhattan plot showing genome-wide distribution from both groups.

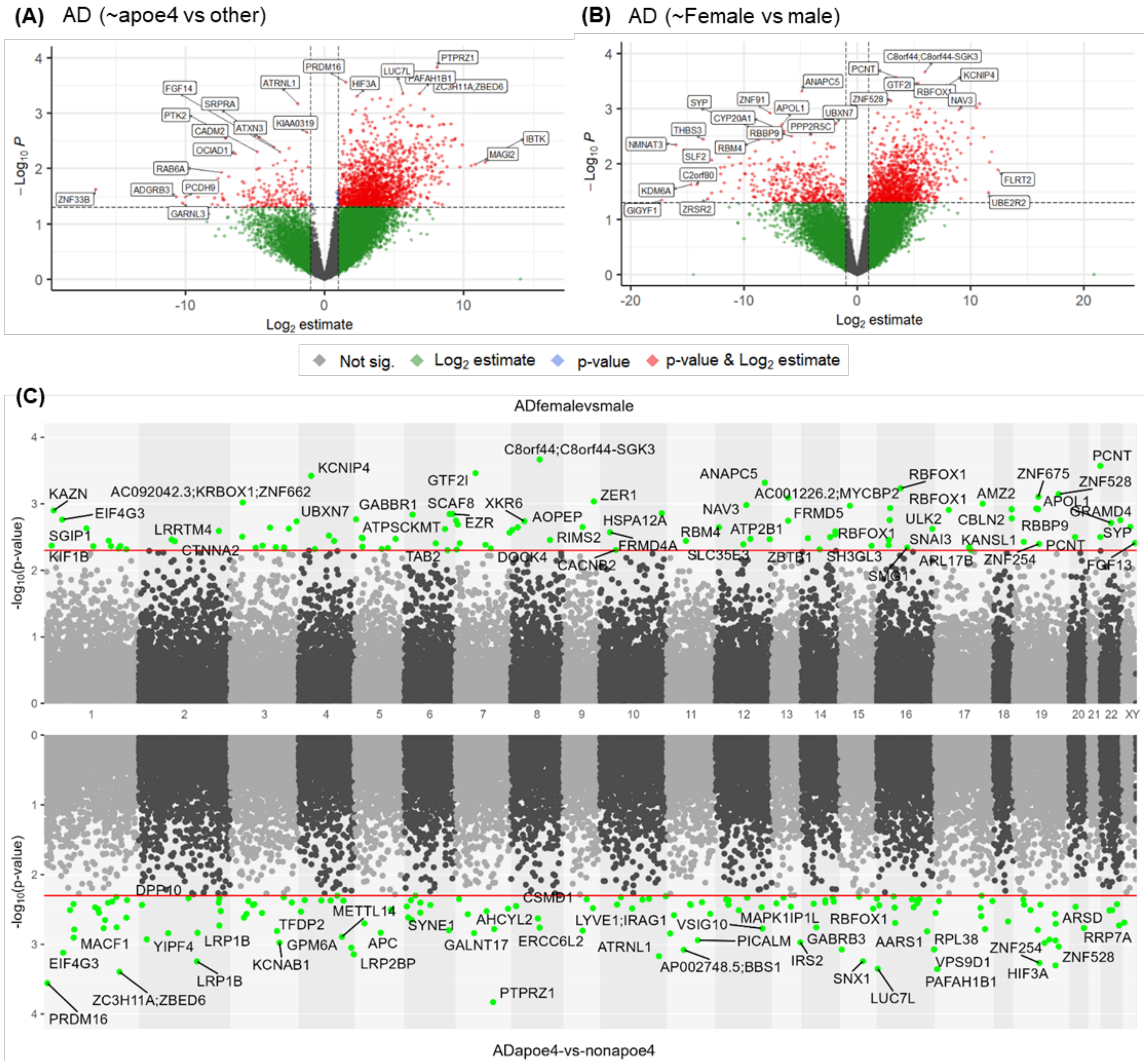

Figure S12. Differential pattern of RNA editing events from IFG brain region, volcano plots showing (A) AD apoe4 vs AD nonapoe4, (B) AD female vs AD male, (C) mirrored Manhattan plot showing genome-wide distribution from both groups.

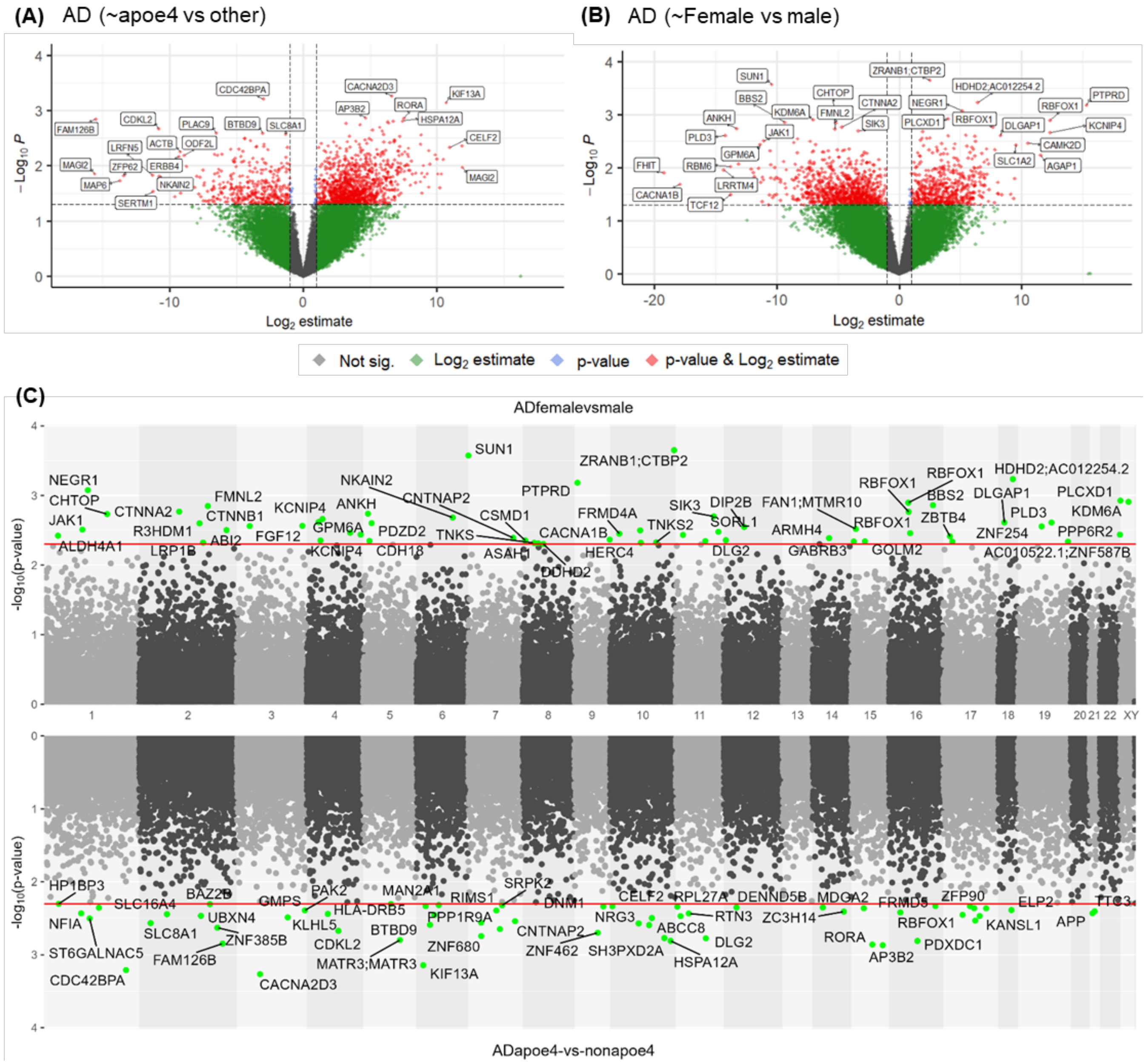

Figure S13. Differential pattern of RNA editing events from STG brain region, volcano plots showing (A) AD apoe4 vs AD nonapoe4, (B) AD female vs AD male, (C) mirrored Manhattan plot showing genome-wide distribution from both groups.

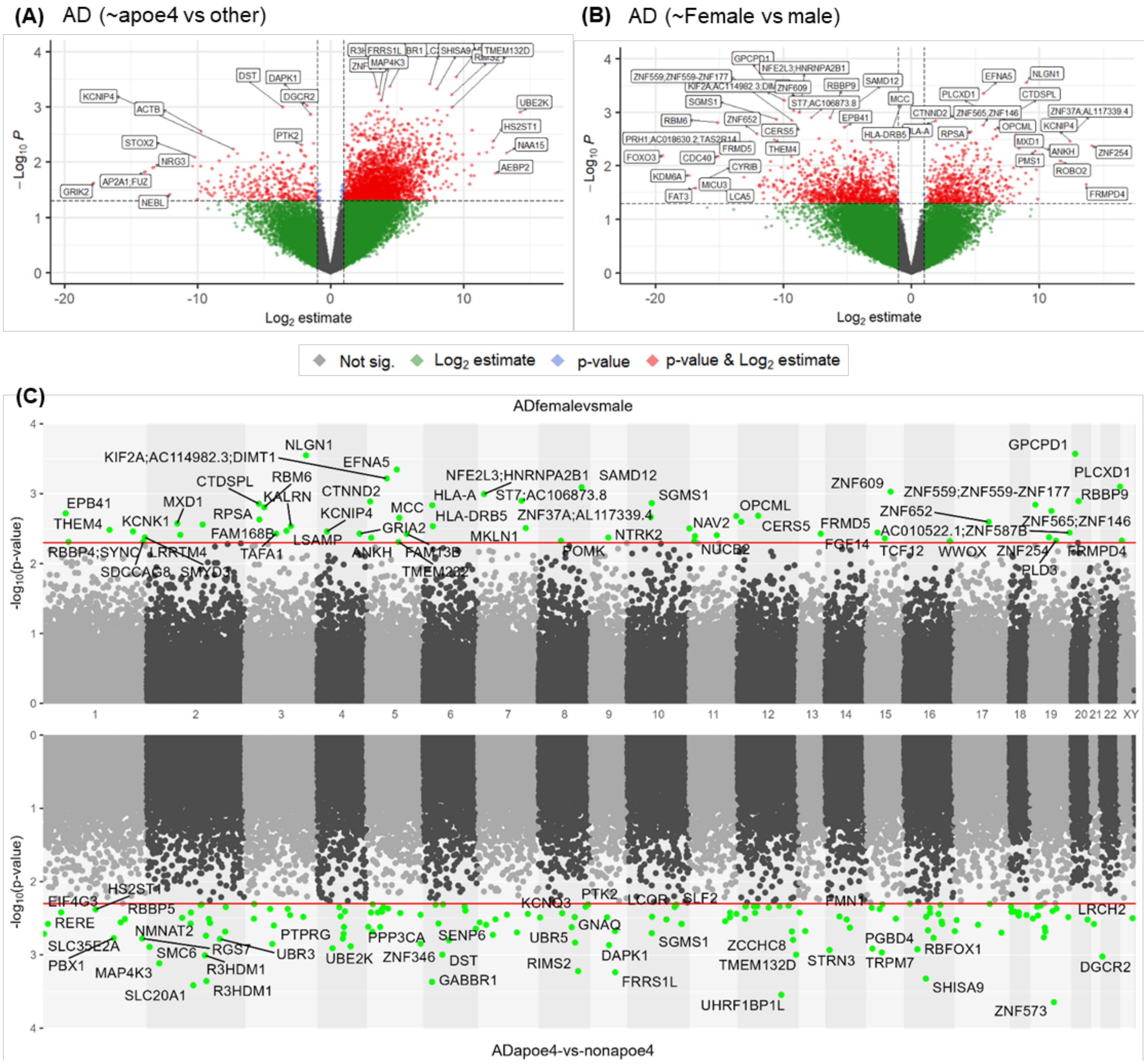

Figure S14. Differential pattern of RNA editing events from FP brain region, volcano plots showing (A) AD apoe4 vs AD nonapoe4, (B) AD female vs AD male, (C) mirrored Manhattan plot showing genome-wide distribution from both groups.

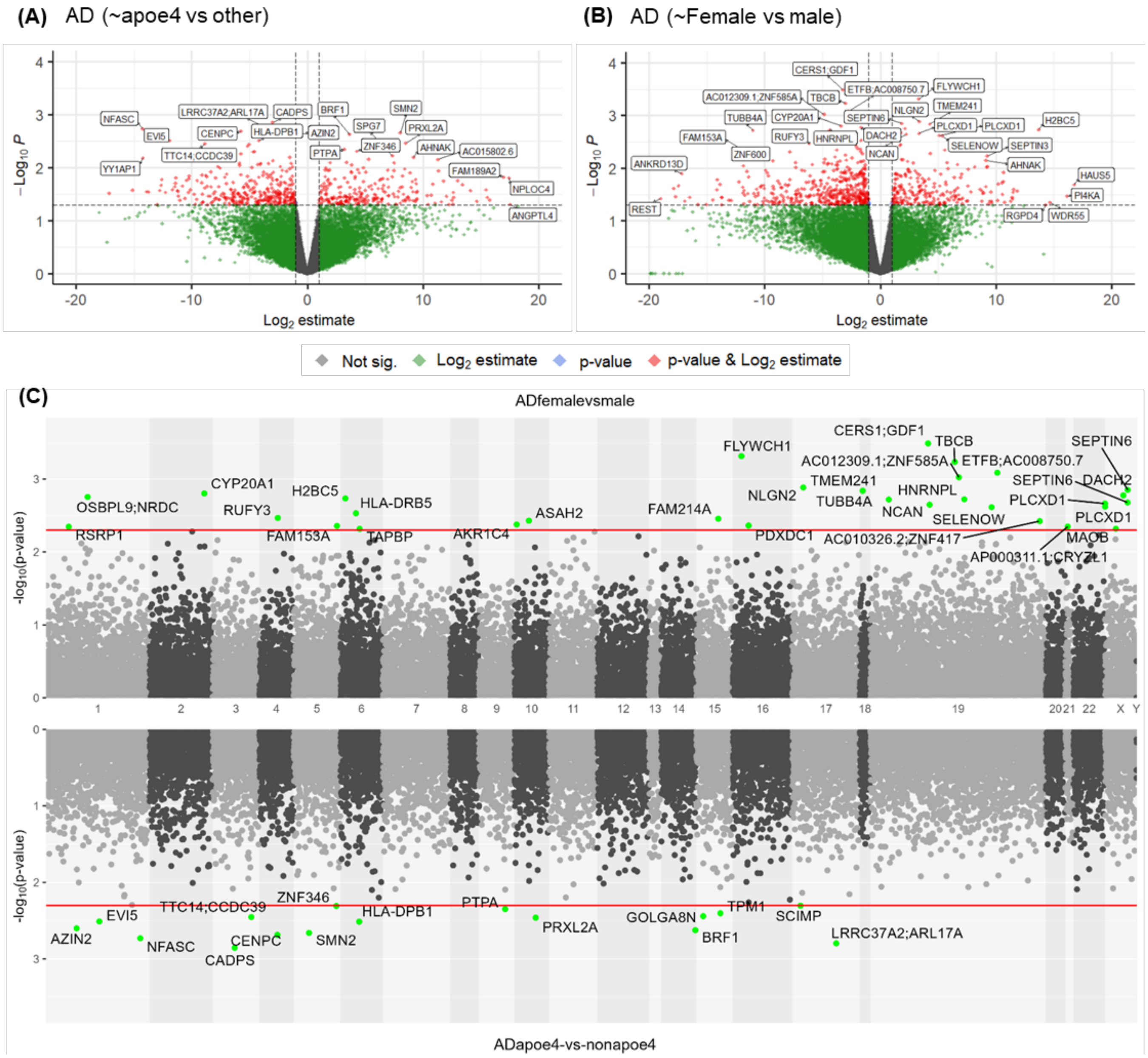

Figure S15. Differential pattern of RNA editing events from TCX brain region, volcano plots showing (A) AD apoe4 vs AD nonapoe4, (B) AD female vs AD male, (C) mirrored Manhattan plot showing genome-wide distribution from both groups.

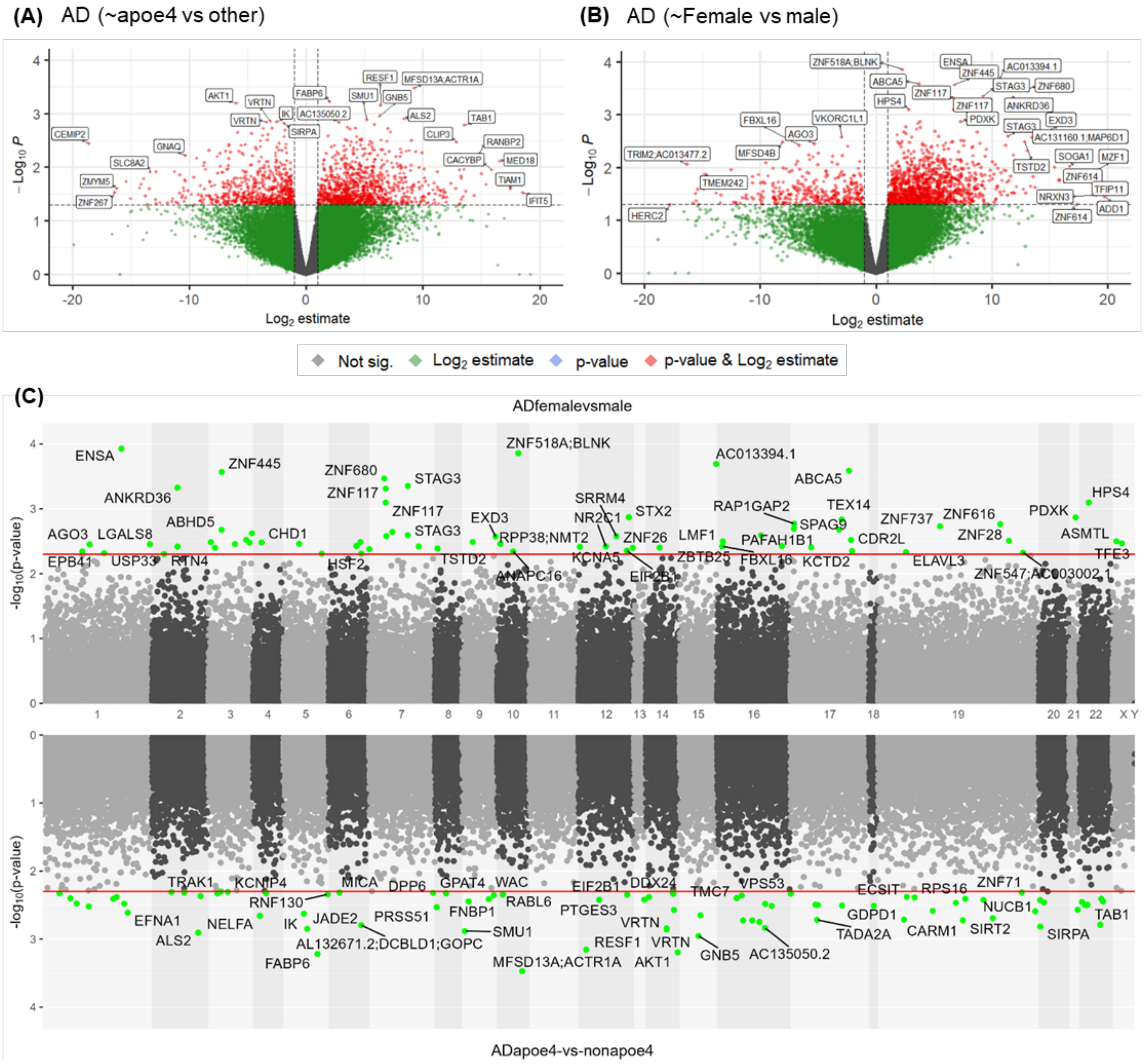

Figure S16. Differential pattern of RNA editing events from CBE brain region, volcano plots showing (A) AD apoe4 vs AD nonapoe4, (B) AD female vs AD male, (C) mirrored Manhattan plot showing genome-wide distribution from both groups.

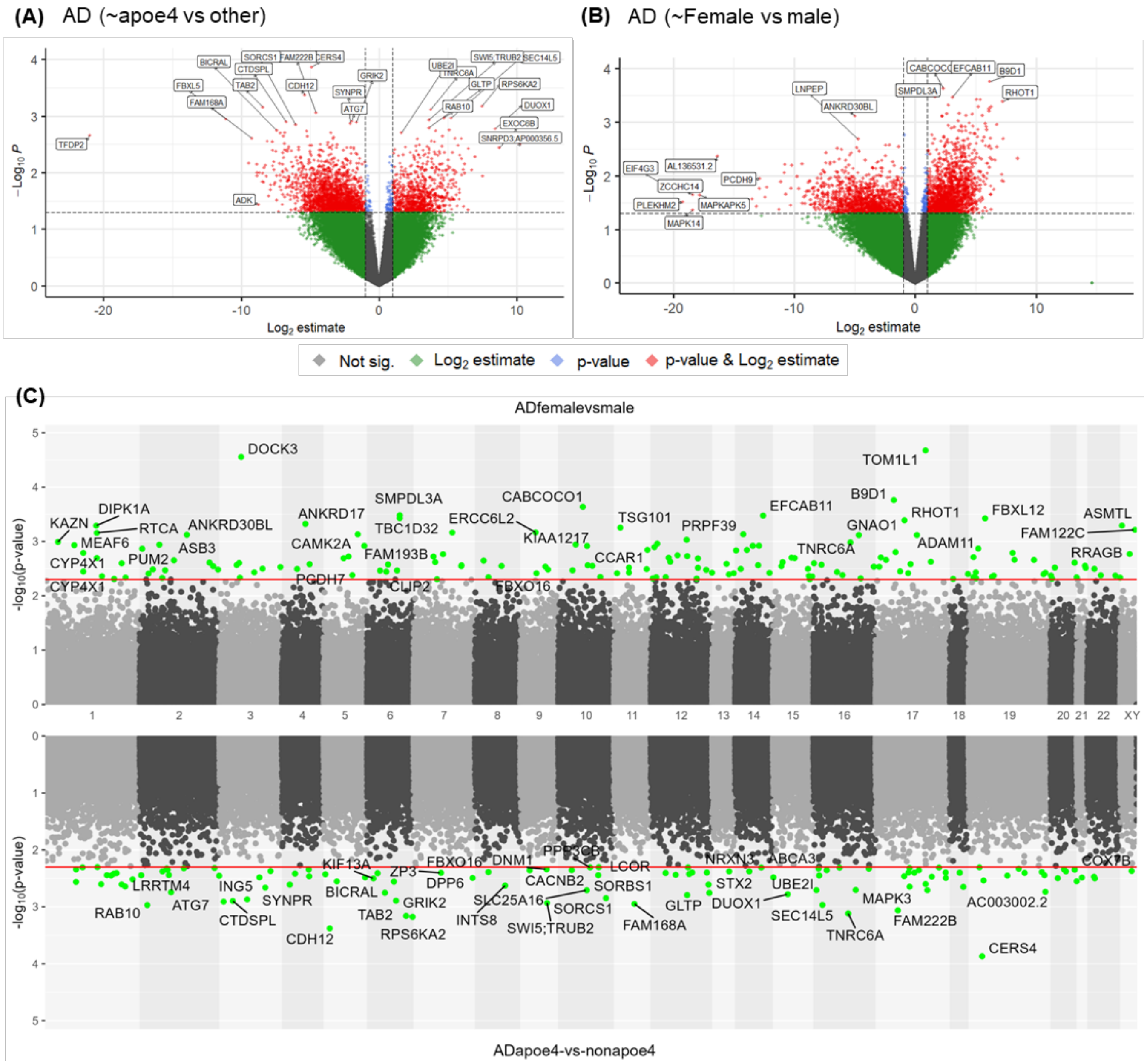

Figure S17. Differential pattern of RNA editing events from DLPFC brain region, volcano plots showing (A) AD apoe4 vs AD nonapoe4, (B) AD female vs AD male, (C) mirrored Manhattan plot showing genome-wide distribution from both groups.

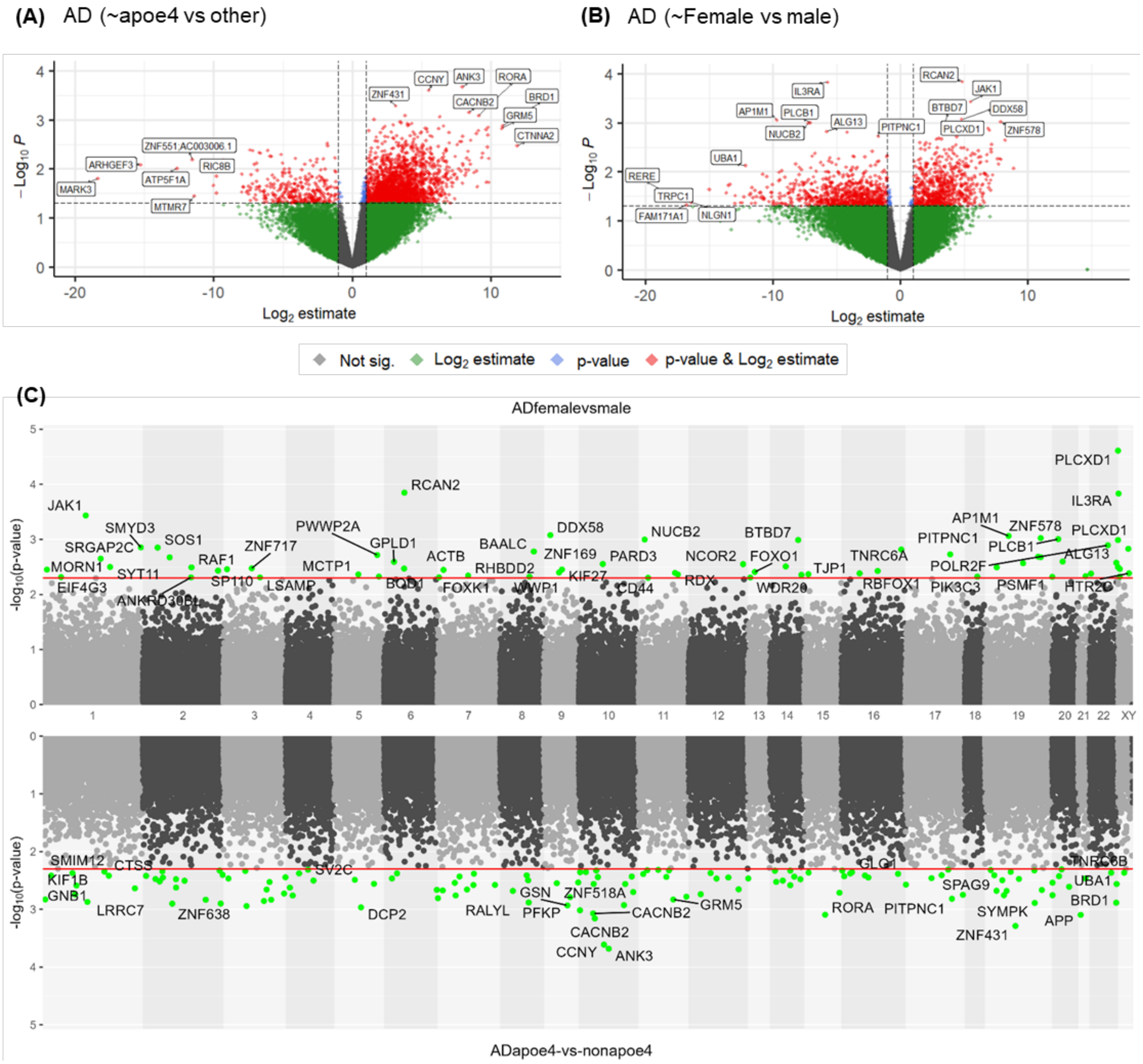

Figure S18. Differential pattern of RNA editing events from ACC brain region, volcano plots showing (A) AD apoe4 vs AD nonapoe4, (B) AD female vs AD male, (C) mirrored Manhattan plot showing genome-wide distribution from both groups.

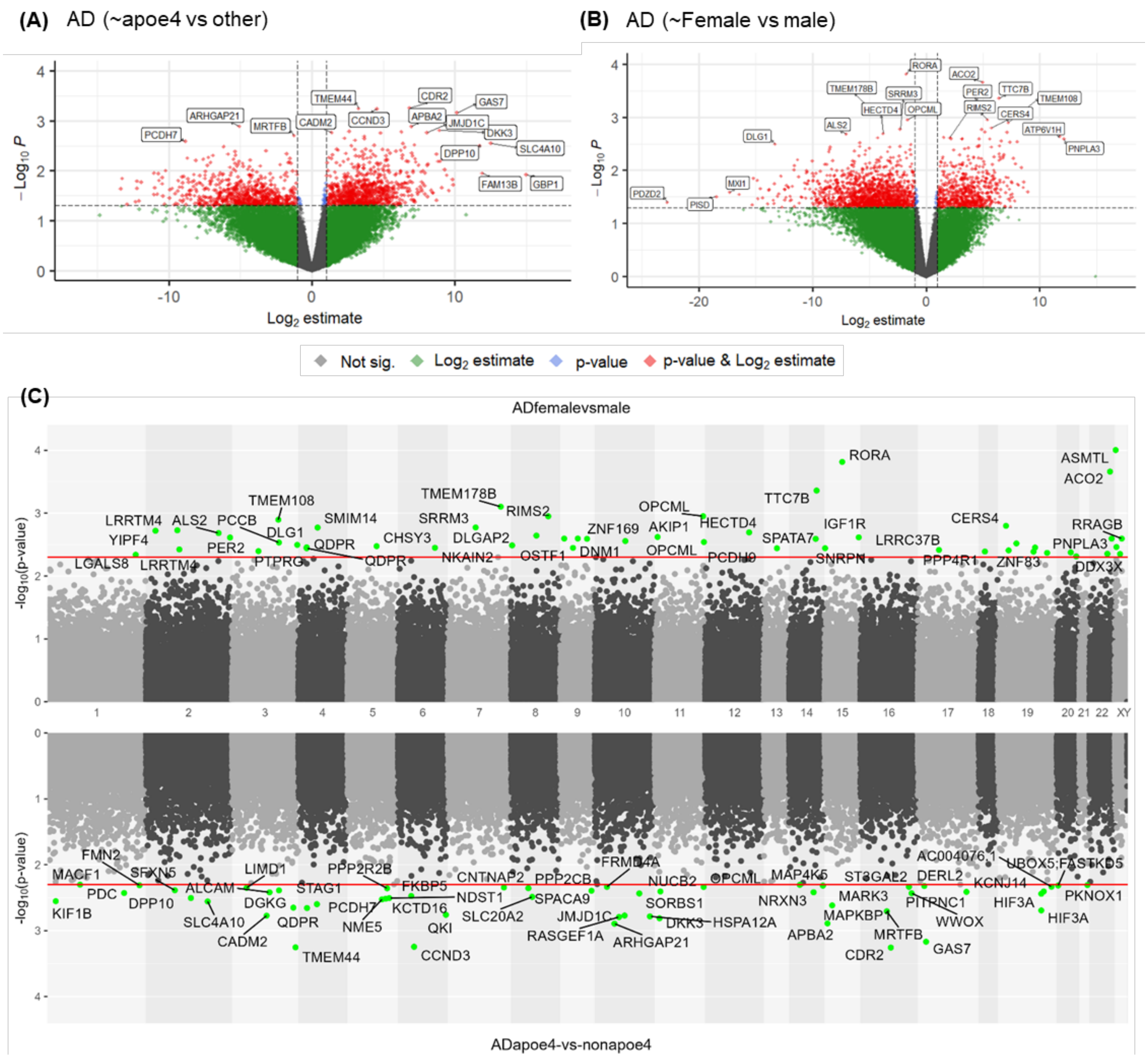

Figure S19. Differential pattern of RNA editing events from PCC brain region, volcano plots showing (A) AD apoe4 vs AD nonapoe4, (B) AD female vs AD male, (C) mirrored Manhattan plot showing genome-wide distribution from both groups.

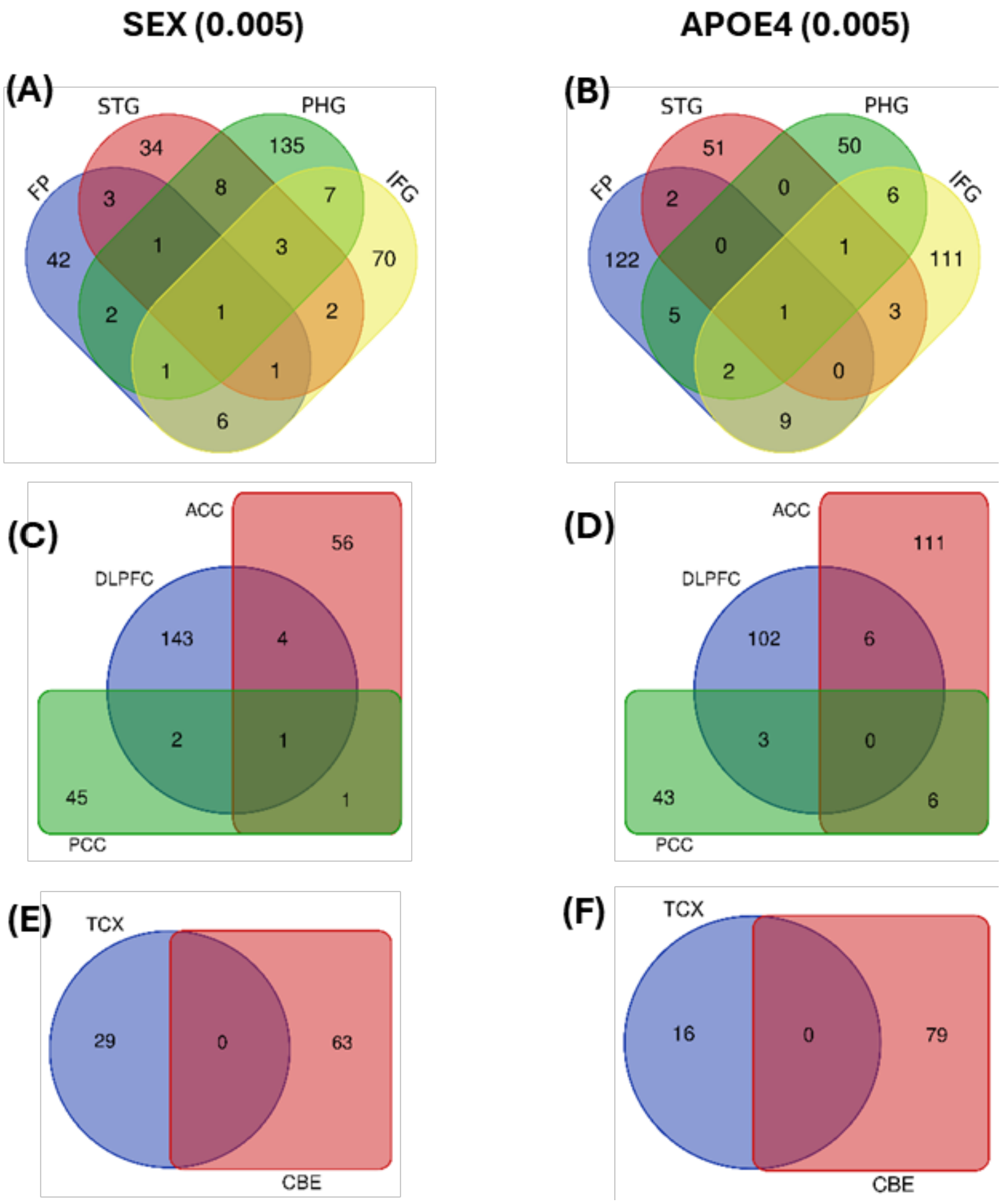

Figure S20. Sex-specific (A, C, E) and APOE4-specifc (B, D, F) RNA edited genes shared among multiple brain regions from three biobanks (A, B) MSBB, (C, D) ROSMAP, (E, F) MAYO.

(A)

Brain regions	*RNAseq samples*	*WGS+RNAseq samples*	*cis-edQTLs*	
			±100 KB	*± 1 MB*
TCX	259	255	16,996	14,732,375
CBE	246	243	24,449	23,128,645
FP	318	302	14,155	7,455,807
PHG	308	292	9,650	9,695,092
STG	324	308	9,748	9,501,019
IFG	297	281	12,236	11,516,527
DLPFC	1092	821	16,170	15,655,105
PCC	647	496	26,475	22,753,150
ACC	717	629	30,386	28,372,523
Total	4208	3627		

(B) STG

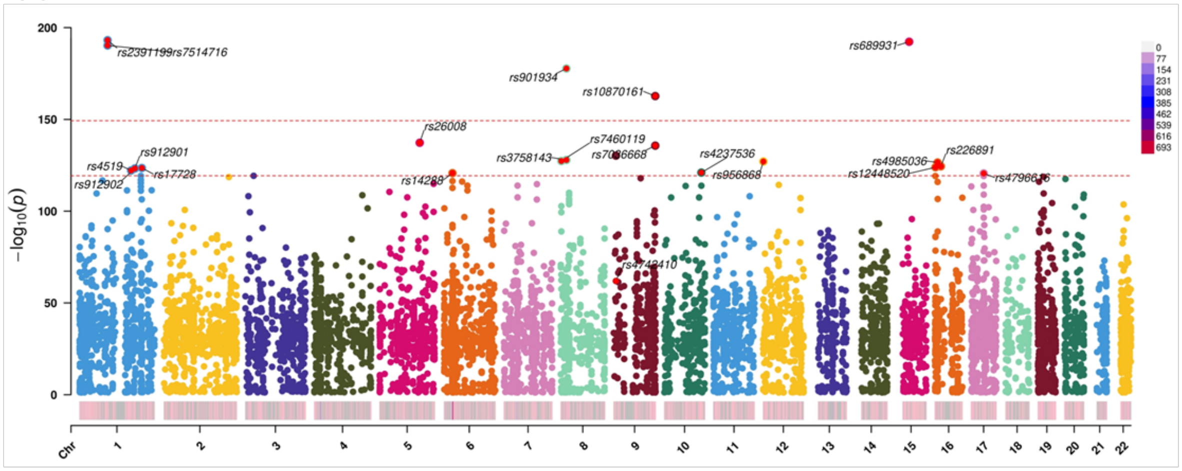

(C) FP

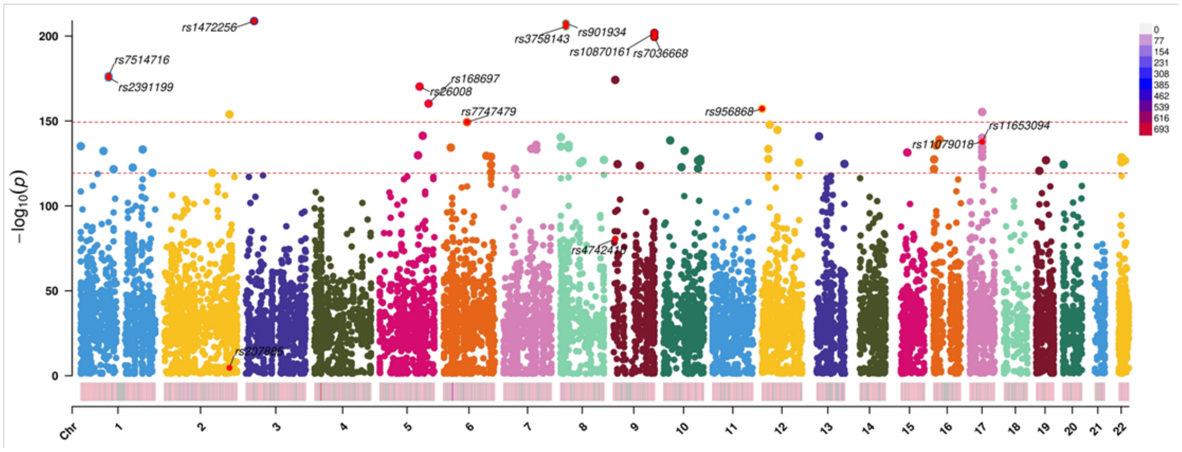

Figure S21. (A) Illustrating number of samples and cis-edQTLs from the nine brain regions within ±100 KB and ±1 MB distance window. Genome-wide distribution of the cis-edQTLs pertaining to tissues from MSBB biobank (B) STG, (C) FP.

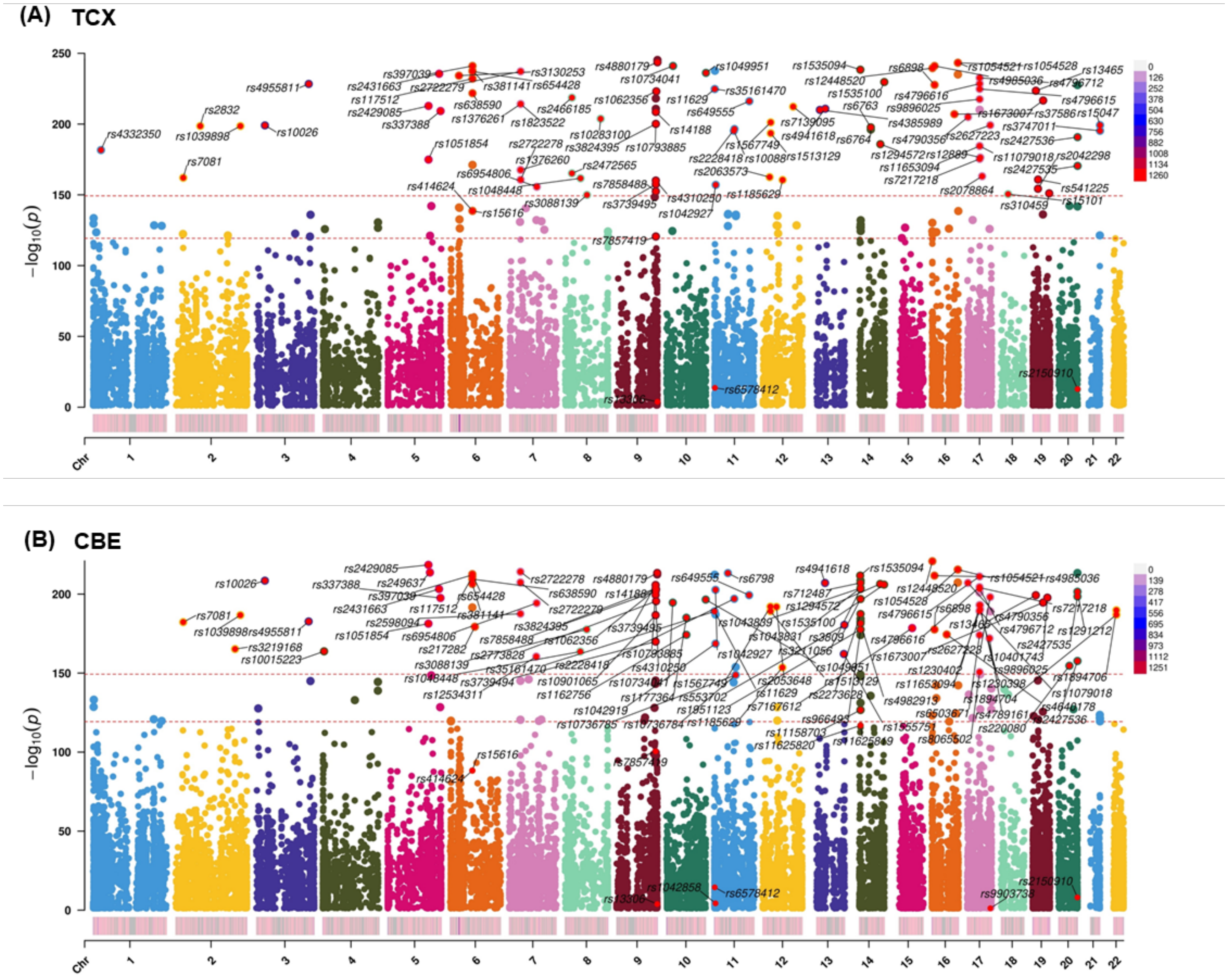

Figure S22. Genome-wide distribution of the cis-edQTLs from individual tissues, (A) TCX, (B) CBE from MAYO biobank.

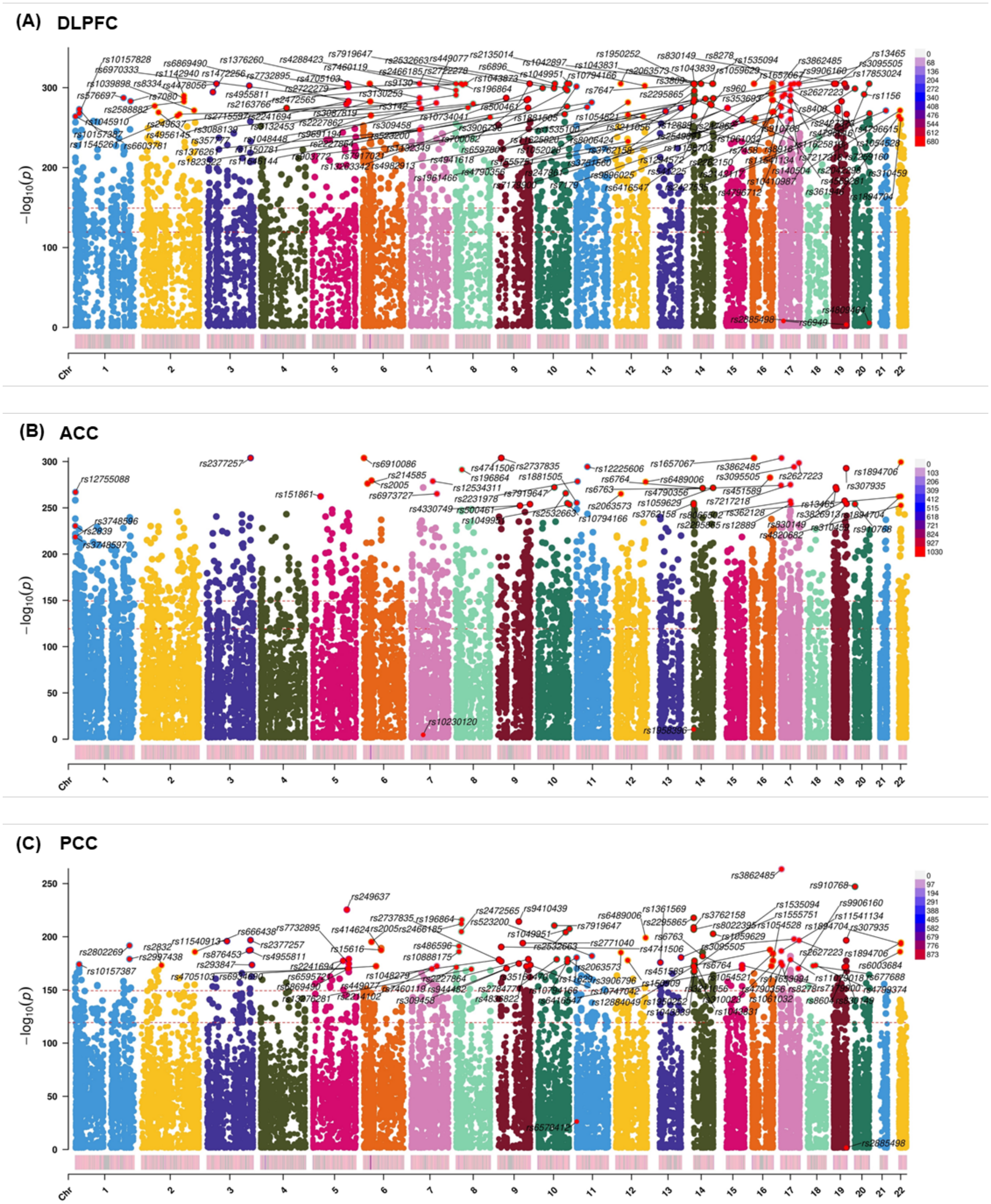

Figure S23. Genome-wide distribution of the cis-edQTLs from distinct tissues, (A) DLPFC, (B) ACC, (C) PCC from ROSMAP biobank.
